# Thoracic and cardiovascular surgeries in Japan during 2022: Annual report by the Japanese Association for Thoracic Surgery

**DOI:** 10.1007/s11748-024-02106-x

**Published:** 2025-02-12

**Authors:** Committee for Scientific Affairs, The Japanese Association for Thoracic Surgery, Naoki Yoshimura, Yukio Sato, Hiroya Takeuchi, Tomonobu Abe, Toyofumi Fengshi Yoshikawa, Yasutaka Hirata, Michiko Ishida, Hisashi Iwata, Takashi Kamei, Nobuyoshi Kawaharada, Shunsuke Kawamoto, Kohji Kohno, Hiraku Kumamaru, Kenji Minatoya, Noboru Motomura, Rie Nakahara, Morihito Okada, Hisashi Saji, Aya Saito, Masanori Tsuchida, Kenji Suzuki, Hirofumi Takemura, Tsuyoshi Taketani, Yasushi Toh, Wataru Tatsuishi, Hiroyuki Yamamoto, Takushi Yasuda, Masayuki Watanabe, Goro Matsumiya, Yoshiki Sawa, Hideyuki Shimizu, Masayuki Chida

**Affiliations:** 1 Committee for Scientific Affairs, The Japanese Association for Thoracic Surgery, Tokyo, Japan; 2 Department of Thoracic and Cardiovascular Surgery, Graduate School of Medicine, University of Toyama, Toyama, Japan; 3 Department of Thoracic Surgery, University of Tsukuba, Tsukuba, Japan; 4 Department of Surgery, Hamamatsu University School of Medicine, Shizuoka, Japan; 5 Division of Cardiovascular Surgery, Department of General Surgical Science, Gunma University, Maebashi, Japan; 6 Department of Thoracic Surgery, Nagoya University Graduate School of Medicine, Nagoya, Japan; 7 Department of Cardiovascular Surgery, National Center for Child Health and Development, Tokyo, Japan; 8 Cardiac Surgery, Handa City Hospita, Aichi, Japan; 9 Department of General Thoracic Surgery, Gifu University Hospital, Gifu, Japan; 10 Department of Surgery, Graduate School of Medicine, Tohoku University, Sendai, Japan; 11 Department of Cardiovascular Surgery, Sapporo Medical University School of Medicine, Sapporo, Japan; 12 Department of Cardiovascular Surgery, Tohoku Medical and Pharmaceutical University Hospital, Sendai, Japan; 13 Department of Gastrointestinal Tract Surgery, Fukushima Medical University, Fukushima, Japan; 14 Department of Healthcare Quality Assessment, Graduate School of Medicine, The University of Tokyo, Tokyo, Japan; 15 Department of Cardiovascular Surgery, Graduate School of Medicine, Kyoto University, Kyoto, Japan; 16 Department of Cardiovascular Surgery, Toho University Sakura Medical Center, Chiba, Japan; 17 Division of Thoracic Surgery, Tochigi Cancer Center, Tochigi, Japan; 18 Surgical Oncology, Hiroshima University, Hiroshima, Japan; 19 Department of Chest Surgery, St. Marianna University School of Medicine, Kawasaki, Japan; 20 Department of Surgery, Graduate School of Medicine, Yokohama City University, Yokohama, Japan; 21 Division of Thoracic and Cardiovascular Surgery, Niigata University Graduate School of Medical and Dental Sciences, Niigata, Japan; 22 Department of General Thoracic Surgery, Juntendo University School of Medicine, Tokyo, Japan; 23 Department of Cardiovascular Surgery, Kanazawa University, Kanazawa, Japan; 24 Department of Cardiovascular Surgery, Mitsui Memorial Hospital, Tokyo, Japan; 25 Department of Gastroenterological Surgery, National Hospital Organization Kyushu Cancer Center, Fukuoka, Japan; 26 Department of Surgery, Faculty of Medicine, Kindai University, Osaka, Japan; 27 Department of Gastroenterological Surgery, Cancer Institute Hospital, Tokyo, Japan; 28 Department of Cardiovascular Surgery, Chiba University Graduate School of Medicine, Chiba, Japan; 29 Graduate School of Medicine, Osaka University, Osaka Police Hospital, Osaka, Japan; 30 Department of Cardiovascular Surgery, Keio University, Tokyo, Japan; 31 Department of General Thoracic Surgery, Dokkyo Medical University, Tochigi, Japan

Since 1986, the Japanese Association for Thoracic Surgery (JATS) has conducted annual thoracic surgery surveys throughout Japan to determine statistics on the number of procedures performed by surgical categories. Herein, we summarize the results of the association’s annual thoracic surgery surveys in 2022.

Adhering to the norm thus far, thoracic surgery had been classified into three categories, including cardiovascular, general thoracic, and esophageal surgeries, with patient data for each group being examined and analyzed. We honor and value all members’ continued professional support and contributions.

Incidence of hospital mortality was included in the survey to determine nationwide status, which has contributed to Japanese surgeons’ understanding of the present status of thoracic surgery in Japan while helping in surgical outcome improvements by enabling comparisons between their work and that of others. This approach has enabled the association to gain a better understanding of present problems and prospects, which is reflected in its activities and member education.

The 30-day mortality (also known as *operative mortality*) is defined as death within 30 days of surgery, regardless of the patient’s geographic location, including post-discharge from the hospital. *Hospital mortality* is defined as death within any time interval following surgery among patients yet to be discharged from the hospital.

Transfer to a nursing home or a rehabilitation unit is considered hospital discharge unless the patient subsequently dies of complications from surgery, while hospital-to-hospital transfer during esophageal surgery is not considered a form of discharge. In contrast, hospital-to-hospital transfer 30 days following cardiovascular and general thoracic surgeries are considered discharge given that National Clinical Database (NCD)-related data were used in these categories.

Severe Acute Respiratory Syndrpme Coronavirus-2 (SARS-CoV-2), the causative pathogen for the coronavirus disease 2019 (COVID-19), first emerged in Wuhan, China, in December 2019 and by March 2020, it was declared a pandemic [1]. The pandemic of SARS-CoV-2 resulted in a global healthcare and financial crisis. There was a significant estimated reduction in national case volume of cardiovascular, general thoracic, and esophageal surgeries in Japan during 2020 [2–5]. We have to continue the estimation of the nationwide effect of SARS-CoV-2 pandemic on thoracic surgery in Japan, with surgical volume, outcomes and patient data for each group.

## Survey abstract

All data on cardiovascular, general thoracic, and esophageal surgeries were obtained from the NCD. In 2018, the data collection method for general thoracic and esophageal surgeries had been modified from self-reports using questionnaire sheets following each institution belonging to the JATS to an automatic package downloaded from the NCD in Japan.

The data collection related to cardiovascular surgery (initially self-reported using questionnaire sheets in each participating institution up to 2014) changed to downloading an automatic package from the Japanese Cardiovascular Surgery Database (JCVSD), which is a cardiovascular subsection of the NCD in 2015.

## Final report: 2022

### (A) Cardiovascular surgery

We are extremely pleased with the cooperation of our colleagues (members) in completing the cardiovascular surgery survey, which has undoubtedly improved the quality of this annual report. We are truly grateful for the significant efforts made by all participants within each participating institution in completing the JCVSD/NCD.

**Fig. 1:**
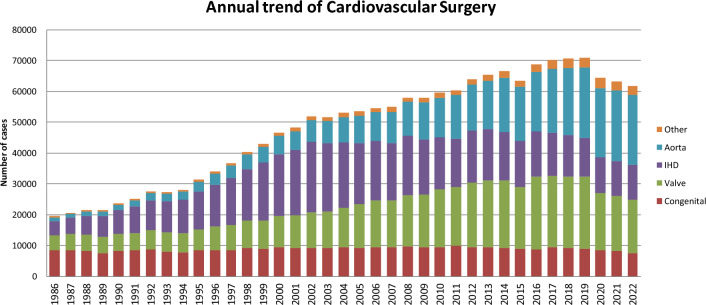
Annual trend of cardiovascular surgery. IHD, ischemic heart disease

Figure 1 illustrates the development of cardiovascular surgery in Japan over the past 35 years. Aortic surgery includes only surgeries for aortic dissection, thoracic and thoracoabdominal aortic aneurysms. Extra-anatomic bypass surgery for thoracic aneurysm and pacemaker implantation have been excluded from the survey since 2015. Ventricular assist device (VAD) implantations had not been included in the total number of surgical procedures but we have decided to count the number of VAD implantation from this time. VAD implantations since 2016 were added to Fig. 1.

A total of 61,606 cardiovascular surgeries, including 120 VAD implantations and 79 heart transplants, had been performed in 2022, with a 2.6% decrease compared to that in 2021 (*n* = 63,198). Following on from 2020, a decline in the number of cases has been observed for the third consecutive year. Although the impact of the COVID-19 pandemic is suggested, verification from various perspectives is necessary.

Compared to data for 2021 [4] and 2012 [6], data for 2022 showed 10.1% (7580 vs. 8349) and 26.1% fewer surgeries for congenital heart disease, 2.3% (17,260 vs. 17,661) and 21.2% fewer surgeries for valvular heart disease, 0.2% (11,340 vs. 11,364) and 47.8% fewer surgeries for ischemic heart procedures, and 1.7% (22,597 vs. 22,982) fewer and 50.0% more surgeries for thoracic aorta, respectively. Data for individual categories are summarized in Tables 1, 2, 3, 4, 5, and 6.

**Table 1:** Congenital (total; 7580) (1) CPB ( +) (total; 5789)

	Neonate				Infant				1–17 years				≥ 18 years				Total			
	Cases	30-day mortality		Hospital mortality	Cases	30-day mortality		Hospital mortality	Cases	30-day mortality		Hospital mortality	Cases	30-day mortality		Hospital mortality	Cases	30-day mortality		Hospital mortality
		Hospital	After discharge			Hospital	After discharge			Hospital	After discharge			Hospital	After discharge			Hospital	After discharge	
PDA	4	0	0	0	1	0	0	0	2	0	0	0	14	0	0	0	21	0	0	0
Coarctation (simple)	4	0	0	0	9	0	0	0	8	0	0	0	7	0	0	0	28	0	0	0
+ VSD	59	0	0	1 (1.7)	54	1 (1.9)	0	2 (3.7)	16	0	0	0	3	0	0	0	132	1 (0.8)	0	3 (2.3)
+ DORV	7	0	0	0	9	0	0	1 (11.1)	3	0	0	0	0	0	0	0	19	0	0	1 (5.3)
+ AVSD	5	1 (20.0)	0	1 (20.0)	5	0	0	0	1	0	0	0	0	0	0	0	11	1 (9.1)	0	1 (9.1)
+ TGA	0	0	0	0	1	0	0	0	0	0	0	0	0	0	0	0	1	0	0	0
+ SV	2	0	0	0	1	0	0	0	1	0	0	0	0	0	0	0	4	0	0	0
+ Others	7	0	0	0	5	0	0	0	2	0	0	0	2	0	0	0	16	0	0	0
Interrupt. of Ao (simple)	0	0	0	0	0	0	0	0	0	0	0	0	0	0	0	0	0	0	0	0
+ VSD	10	0	0	2 (20.0)	19	1 (5.3)	0	1 (5.3)	7	0	0	0	0	0	0	0	36	1 (2.8)	0	3 (8.3)
+ DORV	1	0	0	0	0	0	0	0	0	0	0	0	0	0	0	0	1	0	0	0
+ Truncus	3	1 (33.3)	0	2 (66.7)	7	0	0	1 (14.3)	3	0	0	0	1	0	0	0	14	1 (7.1)	0	3 (21.4)
+ TGA	0	0	0	0	1	0	0	0	0	0	0	0	0	0	0	0	1	0	0	0
+ Others	1	0	0	0	1	0	0	0	2	0	0	0	0	0	0	0	4	0	0	0
Vascular ring	0	0	0	0	2	0	0	0	0	0	0	0	0	0	0	0	2	0	0	0
PS	3	0	0	1 (33.3)	16	0	0	0	51	0	0	1 (2.0)	16	0	0	0	86	0	0	2 (2.3)
PA⋅IVS or Critical PS	8	0	0	0	34	0	0	2 (5.9)	35	0	0	0	6	0	0	0	83	0	0	2 (2.4)
TAPVR	73	3 (4.1)	0	6 (8.2)	56	0	0	0	15	0	0	0	1	0	0	0	145	3 (2.1)	0	6 (4.1)
PAPVR ± ASD	0	0	0	0	3	0	0	0	40	0	0	0	13	0	0	0	56	0	0	0
ASD	0	0	0	0	32	0	0	1 (3.1)	384	1 (0.3)	0	1 (0.3)	777	0	0	1 (0.1)	1,193	1 (0.1)	0	3 (0.3)
Cor triatriatum	1	0	0	1 (100.0)	5	0	0	0	1	0	0	0	1	0	0	0	8	0	0	1 (12.5)
AVSD (partial)	0	0	0	0	3	0	0	0	30	0	0	0	12	0	0	0	45	0	0	0
AVSD (complete)	7	0	0	2 (28.6)	75	1 (1.3)	0	2 (2.7)	88	0	0	1 (1.1)	4	0	0	0	174	1 (0.6)	0	5 (2.9)
+ TOF or DORV	0	0	0	0	2	0	0	0	6	0	0	0	1	0	0	0	9	0	0	0
+ Others	0	0	0	0	0	0	0	0	0	0	0	0	0	0	0	0	0	0	0	0
VSD (subarterial)	5	0	0	0	70	0	0	0	111	0	0	0	12	0	0	0	198	0	0	0
VSD (perimemb./muscular)	7	0	0	0	563	0	0	0	281	0	0	0	22	0	0	0	873	0	0	0
VSD (Type Unknown)	0	0	0	0	0	0	0	0	2	0	0	0	116	2 (1.7)	0	2 (1.7)	118	2 (1.7)	0	2 (1.7)
VSD + PS	0	0	0	0	26	0	0	0	14	0	0	0	0	0	0	0	40	0	0	0
DCRV ± VSD	0	0	0	0	8	0	0	0	16	0	0	0	12	0	0	0	36	0	0	0
Aneurysm of sinus of Valsalva	0	0	0	0	0	0	0	0	0	0	0	0	0	0	0	0	0	0	0	0
TOF	9	0	0	0	173	0	0	0	148	2 (1.4)	0	3 (2.0)	45	0	0	0	375	2 (0.5)	0	3 (0.8)
PA + VSD	6	0	0	0	74	0	0	0	106	1 (0.9)	0	2 (1.9)	8	0	0	0	194	1 (0.5)	0	2 (1.0)
DORV	15	0	0	1 (6.7)	110	0	0	1 (0.9)	124	0	0	1 (0.8)	11	0	0	0	260	0	0	3 (1.2)
TGA (simple)	69	3 (4.3)	0	3 (4.3)	3	0	0	0	1	0	0	0	6	0	0	0	79	3 (3.8)	0	3 (3.8)
+ VSD	24	1 (4.2)	0	2 (8.3)	12	1 (8.3)	0	1 (8.3)	4	0	0	0	1	0	0	0	41	2 (4.9)	0	3 (7.3)
VSD + PS	0	0	0	0	0	0	0	0	0	0	0	0	0	0	0	0	0	0	0	0
Corrected TGA	2	0	0	0	18	1 (5.6)	0	1 (5.6)	24	1 (4.2)	0	1 (4.2)	9	0	0	1 (11.1)	53	2 (3.8)	0	3 (5.7)
Truncus arteriosus	6	0	0	0	13	0	0	1 (7.7)	21	0	0	0	0	0	0	0	40	0	0	1 (2.5)
SV	20	1 (5.0)	0	1 (5.0)	108	4 (3.7)	0	6 (5.6)	139	0	0	0	14	0	0	1 (7.1)	281	5 (1.8)	0	8 (2.8)
TA	8	0	0	0	30	0	0	0	30	0	0	0	3	0	0	0	71	0	0	0
HLHS	25	0	0	4 (16.0)	71	5 (7.0)	0	9 (12.7)	75	0	0	1 (1.3)	0	0	0	0	171	5 (2.9)	0	14 (8.2)
Aortic valve lesion	6	1 (16.7)	0	2 (33.3)	17	0	0	0	79	1 (1.3)	0	2 (2.5)	26	0	0	0	128	2 (1.6)	0	4 (3.1)
Mitral valve lesion	1	0	0	0	20	0	0	0	71	0	0	1 (1.4)	20	3 (15.0)	0	3 (15.0)	112	3 (2.7)	0	4 (3.6)
Ebstein	5	1 (20.0)	0	2 (40.0)	10	0	0	0	17	0	0	0	22	0	0	0	54	1 (1.9)	0	2 (3.7)
Coronary disease	0	0	0	0	11	0	0	1 (9.1)	21	0	0	0	3	0	0	0	35	0	0	1 (2.9)
Others	5	0	0	0	23	1 (4.3)	0	1 (4.3)	44	2 (4.5)	0	2 (4.5)	227	0	0	1 (0.4)	299	3 (1.0)	0	4 (1.3)
Conduit failure	0	0	0	0	0	0	0	0	22	0	0	0	5	0	0	0	27	0	0	0
Redo (excluding conduit failure)	4	0	0	0	40	2 (5.0)	0	3 (7.5)	83	1 (1.2)	0	2 (2.4)	88	2 (2.3)	0	4 (4.5)	215	5 (2.3)	0	9 (4.2)
Total	412	12 (2.9)	0	31 (7.5)	1741	17 (1.0)	0	34 (2.0)	2128	9 (0.4)	0	18 (0.8)	1508	7 (0.5)	0	13 (0.9)	5,789	45 (0.8)	0	96 (1.7)
(), % mortality																				
*CPB* cardiopulmonary bypass; *PDA* patent ductus arteriosus; *VSD* ventricular septal defect; *DORV* double outlet right ventricle; *AVSD* atrioventricular septal defect; *TGA* transposition of great arteries; *SV* single ventricle; *Interrupt. of Ao.* interruption of aorta; *PS* pulmonary stenosis; *PA-IVS* pulmonary atresia with intact ventricular septum; *TAPVR* total anomalous pulmonary venous return; *PAPVR* partial anomalous pulmonary venous return; *ASD* atrial septal defect; *TOF* tetralogy of Fallot; *DCRV* double-chambered right ventricle; *TA* tricuspid atresia; *HLHS* hypoplastic left heart syndrome; *RV-PA* right ventricle-pulmonary artery																				

(2) CPB (−) (total; 1791)																				
	Neonate				Infant				1–17 years				≥ 18 years				Total			
	Cases	30-day mortality		Hospital mortality	Cases	30-day mortality		Hospital mortality	Cases	30-day mortality		Hospital mortality	Cases	30-day mortality		Hospital mortality	Cases	30-day mortality		Hospital mortality
		Hospital	After discharge			Hospital	After discharge			Hospital	After discharge			Hospital	After discharge			Hospital	After discharge	
PDA	227	6 (2.6)	0	9 (4.0)	108	2 (1.9)	0	5 (4.6)	9	0	0	0	1	0	0	0	345	8 (2.3)	0	14 (4.1)
Coarctation (simple)	16	0	0	0	13	0	0	0	4	0	0	0	0	0	0	0	33	0	0	0
+ VSD	46	2 (4.3)	0	3 (6.5)	16	0	0	0	6	0	0	0	0	0	0	0	68	2 (2.9)	0	3 (4.4)
+ DORV	12	0	0	1 (8.3)	4	0	0	0	0	0	0	0	0	0	0	0	16	0	0	1 (6.3)
+ AVSD	4	0	0	0	1	0	0	0	0	0	0	0	0	0	0	0	5	0	0	0
+ TGA	0	0	0	0	0	0	0	0	0	0	0	0	0	0	0	0	0	0	0	0
+ SV	3	0	0	0	1	0	0	0	0	0	0	0	0	0	0	0	4	0	0	0
+ Others	4	0	0	0	3	0	0	0	0	0	0	0	0	0	0	0	7	0	0	0
Interrupt. of Ao (simple)	0	0	0	0	0	0	0	0	0	0	0	0	0	0	0	0	0	0	0	0
+ VSD	17	0	0	1 (5.9)	4	0	0	0	0	0	0	0	0	0	0	0	21	0	0	1 (4.8)
+ DORV	2	0	0	1 (50.0)	0	0	0	0	0	0	0	0	0	0	0	0	2	0	0	1 (50.0)
+ Truncus	10	1 (10.0)	0	2 (20.0)	3	0	0	0	0	0	0	0	0	0	0	0	13	1 (7.7)	0	2 (15.4)
+ TGA	0	0	0	0	0	0	0	0	0	0	0	0	0	0	0	0	0	0	0	0
+ Others	1	0	0	0	1	0	0	0	0	0	0	0	0	0	0	0	2	0	0	0
Vascular ring	3	1 (33.3)	0	1 (33.3)	19	0	0	0	6	0	0	0	0	0	0	0	28	1 (3.6)	0	1 (3.6)
PS	0	0	0	0	2	0	0	0	3	0	0	0	0	0	0	0	5	0	0	0
PA⋅IVS or Critical PS	20	1 (5.0)	0	3 (15.0)	22	2 (9.1)	0	2 (9.1)	3	1 (33.3)	0	1 (33.3)	3	0	0	0	48	4 (8.3)	0	6 (12.5)
TAPVR	19	2 (10.5)	0	3 (15.8)	4	0	0	1 (25.0)	0	0	0	0	0	0	0	0	23	2 (8.7)	0	4 (17.4)
PAPVR ± ASD	0	0	0	0	0	0	0	0	0	0	0	0	0	0	0	0	0	0	0	0
ASD	3	0	0	0	0	0	0	0	5	1 (20.0)	0	1 (20.0)	2	0	0	0	10	1 (10.0)	0	1 (10.0)
Cor triatriatum	0	0	0	0	1	0	0	0	0	0	0	0	0	0	0	0	1	0	0	0
AVSD (partial)	1	0	0	0	2	0	0	0	1	0	0	0	0	0	0	0	4	0	0	0
AVSD (complete)	36	0	0	1 (2.8)	48	1 (2.1)	0	2 (4.2)	8	0	0	1 (12.5)	2	0	0	0	94	1 (1.1)	0	4 (4.3)
+ TOF or DORV	0	0	0	0	1	0	0	0	1	0	0	0	1	0	0	0	3	0	0	0
+ Others	0	0	0	0	0	0	0	0	0	0	0	0	0	0	0	0	0	0	0	0
VSD (subarterial)	3	0	0	0	4	0	0	0	0	0	0	0	0	0	0	0	7	0	0	0
VSD (perimemb./muscular)	56	1 (1.8)	0	2 (3.6)	120	0	0	1 (0.8)	7	0	0	0	0	0	0	0	183	1 (0.5)	0	3 (1.6)
VSD (Type Unknown)	0	0	0	0	0		0	0	0	0	0	0	0	0	0	0	0	0	0	0
VSD + PS	1	0	0	0	1	0	0	0	0	0	0	0	0	0	0	0	2	0	0	0
DCRV ± VSD	0	0	0	0	0	0	0	0	0	0	0	0	3	0	0	0	3	0	0	0
Aneurysm of sinus of Valsalva	0	0	0	0	0	0	0	0	0	0	0	0	0	0	0	0	0	0	0	0
TOF	13	0	0	0	50	0	0	0	4	1 (25.0)	0	1 (25.0)	2	0	0	0	69	1 (1.4)	0	1 (1.4)
PA + VSD	9	0	0	0	35	2 (5.7)	0	2 (5.7)	9	0	0	1 (11.1)	1	0	0	0	54	2 (3.7)	0	3 (5.6)
DORV	49	1 (2.0)	0	2 (4.1)	60	0	0	1 (1.7)	4	0	0	1 (25.0)	0	0	0	0	113	1 (0.9)	0	4 (3.5)
TGA (simple)	14	0	0	0	4	0	0	0	0	0	0	0	4	0	0	0	22	0	0	0
+ VSD	5	1 (20.0)	0	2 (40.0)	4	0	0	0	1	0	0	0	0	0	0	0	10	1 (10.0)	0	2 (20.0)
VSD + PS	0	0	0	0	0	0	0	0	0	0	0	0	0	0	0	0	0	0	0	0
Corrected TGA	5	0	0	0	14	0	0	0	10	1 (10.0)	0	1 (10.0)	4	0	0	0	33	1 (3.0)	0	1 (3.0)
Truncus arteriosus	17	0	0	2 (11.8)	7	0	0	0	0	0	0	0	0	0	0	0	24	0	0	2 (8.3)
SV	46	4 (8.7)	0	4 (8.7)	25	1 (4.0)	0	3 (12.0)	20	0	0	0	7	0	0	2 (28.6)	98	5 (5.1)	0	9 (9.2)
TA	14	0	0	0	17	0	0	0	8	0	0	0	1	0	0	0	40	0	0	0
HLHS	65	3 (4.6)	0	11 (16.9)	34	4 (11.8)	0	5 (14.7)	12	0	0	1 (8.3)	0	0	0	0	111	7 (6.3)	0	17 (15.3)
Aortic valve lesion	7	1 (14.3)	0	2 (28.6)	6	0	0	0	10	0	0	0	1	0	0	0	24	1 (4.2)	0	2 (8.3)
Mitral valve lesion	3	0	0	0	9	0	1 (11.1)	0	1	0	0	0	0	0	0	0	13	0	1 (7.7)	0
Ebstein	9	1 (11.1)	0	1 (11.1)	2	1 (50.0)	0	1 (50.0)	5	0	0	0	0	0	0	0	16	2 (12.5)	0	2 (12.5)
Coronary disease	1	0	0	0	1	0	0	0	1	0	0	0	0	0	0	0	3	0	0	0
Others	10	2 (20.0)	0	2 (20.0)	6	2 (33.3)	0	2 (33.3)	8	1 (12.5)	0	1 (12.5)	1	0	0	0	25	5 (20.0)	0	5 (20.0)
Conduit failure	0	0	0	0	0	0	0	0	0	0	0	0	0	0	0	0	0	0	0	0
Redo (excluding conduit failure)	19	0	0	1 (5.3)	70	0	0	2 (2.9)	104	2 (1.9)	0	3 (2.9)	16	1 (6.3)	0	2 (12.5)	209	3 (1.4)	0	8 (3.8)
Total	770	27 (3.5)	0	54 (7.0)	722	15 (2.1)	1 (0.1)	27 (3.7)	250	7 (2.8)	0	12 (4.8)	49	1 (2.0)	0	4 (8.2)	1,791	50 (2.8)	1 (0.06)	97 (5.4)
(), % mortality																				
*CPB* cardiopulmonary bypass; *PDA* patent ductus arteriosus; *VSD* ventricular septal defect; *DORV* double outlet right ventricle; *AVSD* atrioventricular septal defect; *TGA* transposition of the great arteries; *SV* single ventricle; *Interrupt. of Ao*. interruption of aorta; *PS* pulmonary stenosis; *PA-IVS* pulmonary atresia with intact ventricular septum; *TAPVR* total anomalous pulmonary venous return; *PAPVR* partial anomalous pulmonary venous return; *ASD* atrial septal defect; *TOF* tetralogy of Fallot; *DCRV* double-chambered right ventricle; *TA* tricuspid atresia; *HLHS* hypoplastic left heart syndrome; *RV-PA* right ventricle-pulmonary artery																				

(3) Main procedure																					
		Neonate				Infant				1- 17 years				≥ 18 years				Total			
		Cases	30-day mortality			Cases	30-day mortality			Cases	30-day mortality			Cases	30-day mortality			Cases	30-day mortality		
				After discharge	Hospital mortality		Hospital	After discharge	Hospital mortality		Hospital	After discharge	Hospital mortality		Hospital	After discharge	Hospital mortality		Hospital	After discharge	Hospital mortality
1	SP Shunt	94	0	0	1 (1.1)	235	3 (1.3)	0	6 (2.6)	33	1 (3.0)	0	1 (3.0)	0	0	0	0	362	4 (1.1)	0	8 (2.2)
2	PAB	234	4 (1.7)	0	10 (4.3)	244	2 (0.8)	1 (0.4)	6 (2.5)	11	0	0	0	1	0	0	0	490	6 (1.2)	1 (0.2)	16 (3.3)
3	Bidirectional Glenn or hemi-Fontan ± α	0	0	0	0	167	1 (0.6)	0	2 (1.2)	57	0	0	0	1	0	0	0	225	1 (0.4)	0	2 (0.9)
4	Damus-Kaye-Stansel operation	0	0	0	0	13	0	0	0	8	1 (12.5)	0	1 (12.5)	1	0	0	0	22	1 (4.5)	0	1 (4.5)
5	PA reconstruction/repair (including redo)	12	0	0	1 (8.3)	144	0	0	2 (1.4)	175	1 (0.6)	0	1 (0.6)	15	0	0	0	346	1 (0.3)	0	4 (1.2)
6	RVOT reconstruction/repair	8	0	0	0	207	2 (1.0)	0	2 (1.0)	236	1 (0.4)	0	2 (0.8)	34	0	0	0	485	3 (0.6)	0	4 (0.8)
7	Rastelli procedure	2	0	0	0	51	0	0	0	96	0	0	2 (2.1)	5	0	0	0	154	0	0	2 (1.3)
8	Arterial switch procedure	112	4 (3.6)	0	5 (4.5)	18	1 (5.6)	0	1 (5.6)	5	0	0	0	0	0	0	0	135	5 (3.7)	0	6 (4.4)
9	Atrial switch procedure	0	0	0	0	1	0	0	0	2	0	0	0	1	0	0	0	4	0	0	0
10	Double switch procedure	0	0	0	0	4	1 (25.0)	0	1 (25.0)	8	0	0	0	0	0	0	0	12	1 (8.3)	0	1 (8.3)
11	Repair of anomalous origin of CA	0	0	0	0	5	0	0	0	3	0	0	0	2	0	0	0	10	0	0	0
12	Closure of coronary AV fistula	2	0	0	0	1	0	0	0	4	0	0	0	1	0	0	0	8	0	0	0
13	Fontan/TCPC	0	0	0	0	0	0	0	0	262	0	0	0	27	0	0	0	289	0	0	0
14	Norwood procedure	27	2 (7.4)	0	4 (14.8)	79	6 (7.6)	0	10 (12.7)	5	0	0	1 (20.0)	0	0	0	0	111	8 (7.2)	0	15 (13.5)
15	Ventricular septation	0	0	0	0	1	0	0	0	0	0	0	0	0	0	0	0	1	0	0	0
16	Left side AV valve repair (including Redo)	4	0	0	2 (50.0)	24	0	0	0	77	0	0	0	17	0	0	0	122	0	0	2 (1.6)
17	Left side AV valve replace (including Redo)	0	0	0	0	6	0	0	0	37	0	0	2 (5.4)	22	2 (9.1)	0	2 (9.1)	65	2 (3.1)	0	4 (6.2)
18	Right side AV valve repair (including Redo)	9	1 (11.1)	0	3 (33.3)	75	3 (4.0)	0	4 (5.3)	81	0	0	1 (1.2)	71	0	0	0	236	4 (1.7)	0	8 (3.4)
19	Right side AV valve replace (including Redo)	0	0	0	0	0	0	0	0	7	0	0	0	35	1 (2.9)	0	2 (5.7)	42	1 (2.4)	0	2 (4.8)
20	Common AV valve repair (including Redo)	1	0	0	0	15	2 (13.3)	0	3 (20.0)	11	0	0	0	3	0	0	0	30	2 (6.7)	0	3 (10.0)
21	Common AV valve replace (including Redo)	1	0	0	1 (100.0)	6	0	0	1 (16.7)	10	0	0	0	3	0	0	1 (33.3)	20	0	0	3 (15.0)
22	Repair of supra-aortic stenosis	1	0	0	0	6	0	0	1 (16.7)	19	0	0	0	1	1 (100.0)	0	1 (100.0)	27	1 (3.7)	0	2 (7.4)
23	Repair of subaortic stenosis (including Redo)	1	0	0	0	6	0	0	0	25	0	0	0	3	0	0	0	35	0	0	0
24	Aortic valve plasty ± VSD Closure	6	1 (16.7)	0	1 (16.7)	20	0	0	0	24	1 (4.2)	0	1 (4.2)	1	0	0	0	51	2 (3.9)	0	2 (3.9)
25	Aortic valve replacement	0	0	0	0	0	0	0	0	25	0	0	0	34	0	0	0	59	0	0	0
26	AVR with annular enlargement	0	0	0	0	0	0	0	0	11	0	0	0	0	0	0	0	11	0	0	0
27	Aortic root Replace (except Ross)	0	0	0	0	0	0	0	0	10	0	0	1 (10.0)	16	0	0	0	26	0	0	1 (3.8)
28	Ross procedure	0	0	0	0	3	0	0	0	14	0	0	0					17	0	0	0
29	Bilateral pulmonary artery banding	189	11 (5.8)	0	30 (15.9)	17	0	0	0	0	0	0	0	0	0	0	0	206	11 (5.3)	0	30 (14.6)
Total	703	23 (3.3)	0	58 (8.3)	1348	21 (1.6)	1 (0.1)	39 (2.9)	1,256	5 (0.4)	0	13 (1.0)	294	4 (1.4)	0	6 (2.0)	3601	53 (1.5)	1 (0.03)	116 (3.2)	
(), % mortality																					
*SP* systemic-pulmonary; *PAB* pulmonary artery banding; *PA* pulmonary artery; *RVOT* right ventricular outflow tract; *CA* coronary artery; *AV fistula* arteriovenous fistula; *TCPC* total cavopulmonary connection; *AV valve* atrioventricular valve; *VSD* ventricular septal defect; *AVR* aortic valve replacement

**Table 2:** Acquired (total, (1) + (2) + (4) + (5) + (6) + (7) + isolated operations for arrhythmia in (3); 31,044

(1) Valvelar heart disease (total; 17,260)																	
	Valve	Cases	Operation					30-day mortality				Hospital mortality		Redo			
			Mechanical	Bioprosthesis	Repair	Unknown	With CABG	Hospital		After discharge				Cases	30-day mortality		Hospital mortality
								Replace	Repair	Replace	Repair	Replace	Repair		Hosipital	After discharge	
Isolated	A	7834	866	6555	88	325	1685	112 (1.5)	1 (1.1)	2 (0.03)	0	184 (2.5)	1 (1.1)	620	24 (3.9)	0	30 (4.8)
	M	4708	362	890	3422	34	506	51 (4.1)	23 (0.7)	0	0	92 (7.3)	34 (1.1)	574	16 (2.8)	0	28 (4.9)
	T	205	3	48	154	0	32	2 (3.9)	3 (1.9)	0	0	2 (3.9)	8 (5.2)	51	2 (3.9)	0	4 (7.8)
	P	16	0	16	0	0	1	0	0	0	0	0	0	12	0	0	0
A + M		974					151	45 (4.6)		0		73 (7.5)		138	9 (6.5)	0	16 (11.6)
	A		161	761	19	33											
	M		119	356	491	8											
A + T		301					43	9 (3.0)		0		16 (5.3)		39	2 (5.1)	0	3 (7.7)
	A		29	258	4	10											
	T		0	0	294	7											
M + T		2528					246	40 (1.6)		0		75 (3.0)		271	9 (3.3)	0	18 (6.6)
	M		177	795	1549	7											
	T		6	23	2484	16											
A + M + T		638					80	25 (3.9)		0		41 (6.4)		90	8 (8.9)	0	11 (12.2)
	A		63	552	5	18											
	M		55	272	306	5											
	T		0	7	626	5											
Others		56					2	1 (1.8)		0		3 (5.4)		12	1 (8.3)	0	2 (16.7)
Total		17,260					2746	285 (1.7)		2 (0.1)		486 (2.8)		1807	71 (3.9)	0	112 (6.2)

TAVR	Cases	30-day mortality	
	13,534	168	(1.2)

(2) Ischemic heart disease (total, (A) + (B); 11,340)																					
(A) Isolated CABG (total; (a) + (b); 10,226)																					
(a-1) On-pump arrest CABG (total;2372)																					
	Primary, elective				Primary, emergent				Redo, elective				Redo, emergent				Artery only	Artery + SVG	SVG only	Others	Unclear
	Cases	30 day mortality		Hospital mortality	Cases	30 day mortality		Hospital mortality	Cases	30 day mortality		Hospital mortality	Cases	30 day mortality		Hospital mortality					
		Hospital	After discharge			Hospital	after discharge			Hospital	After discharge			Hospital	After discharge						
1VD	64	0	0	1 (1.6)	10	0	0	1 (10.0)	0	0	0	0	3	1 (33.3)	0	1 (33.3)	27	30	19	1	0
2VD	266	5 (1.9)	0	5 (1.9)	28	1 (3.6)	0	3 (10.7)	1	0	0	0	0	0	0	0	42	234	18	1	0
3VD	915	7 (0.8)	0	13 (1.4)	75	8 (10.7)	0	11 (14.7)	1	0	0	0	0	0	0	0	55	902	27	7	0
LMT	816	7 (0.9)	0	12 (1.5)	150	14 (9.3)	0	19 (12.7)	7	0	0	1 (14.3)	5	1 (20.0)	0	2 (40.0)	64	866	44	4	0
no info	17	0	0	1 (5.9)	13	1 (7.7)	0	1 (7.7)	1	0	0	0	0	0	0	0	5	14	9	2	1
Total	2078	19 (0.9)	0	32 (1.5)	276	24 (8.7)	0	35 (12.7)	10	0	0	1 (10.0)	8	2 (25.0)	0	3 (37.5)	193	2046	117	15	1
Kawasaki	6	0	0	0	1	0	0	0	0	0	0	0	1	0	0	0	4	4	0	0	0
On dialysis	257	8 (3.1)	0	12 (4.7)	37	4 (10.8)	0	7 (18.9)	1	0	0	0	3	1 (33.3)	0	1 (33.3)	21	256	20	1	0
(), % mortality																					
*CABG* coronary artery bypass grafting; *1VD* one-vessel disease; *2VD* two-vessel disease; *3VD* three-vessel disease; *LMT* left main trunk; *SVG* saphenous vein graft																					
LMT includes LMT alone or LMT with other branch diseases																					

(a-2) On-pump beating CABG (total; 2034)																					
	Primary, elective				Primary, emergent				Redo, elective				Redo, emergent				Artery only	Artery + SVG	SVG only	others	Unclear
	Cases	30 day mortality		Hospital mortality	Cases	30 day mortality		Hospital mortality	Cases	30 day mortality		Hospital mortality	Cases	30 day mortality		Hospital mortality					
		Hospital	After discharge			Hospital	After discharge			Hospital	After discharge			Hospital	After discharge						
1VD	39	0	0	1 (2.6)	12	3 (25.0)	0	4 (33.3)	6	0	0	0	0	0	0	0	21	22	14	0	0
2VD	198	5 (2.5)	0	9 (4.5)	33	5 (15.2)	0	6 (18.2)	2	0	0	0	0	0	0	0	54	164	15	0	0
3VD	729	11 (1.5)	1 (0.1)	21 (2.9)	122	6 (4.9)	0	11 (9.0)	2	0	0	0	3	0	0	0	103	727	21	5	0
LMT	650	12 (1.8)	0	24 (3.7)	185	15 (8.1)	0	21 (11.4)	8	0	0	0	6	3 (50.0)	0	3 (50.0)	129	679	35	6	0
no info	22	1 (4.5)	0	1 (4.5)	14	4 (28.6)	0	5 (35.7)	2	0	0	0	1	0	0	0	12	17	9	0	1
Total	1638	29 (1.8)	1 0.1	56 (3.4)	366	33 (9.0)	0	47 (12.8)	20	0	0	0	10	3 (30.0)	0	3 (30.0)	319	1609	94	11	1
Kawasaki	1	0	0	0 (0.0)	0	0	0	0	0	0	0	0	0	0	0	0	0	1	0	0	0
on dialysis	228	10 (4.4)	0	23 (10.1)	61	11 (18.0)	0	16 (26.2)	5	0	0	0	4	2 (50.0)	0	2 (50.0)	24	247	23	3	1
(), % mortality																					
*CABG* coronary artery bypass grafting; *1VD* one-vessel disease; *2VD* two-vessel disease; *3VD* three-vessel disease; *LMT* left main trunk; *SVG* saphenous vein graft																					
LMT includes LMT alone or LMT with other branch diseases																					

(b) Off-pump CABG (total; 5820)																					
(Including cases of planned off-pump CABG in which, during surgery, the change is made to an on-pump CABG or on-pump beating-heart procedure)																					
	Primary, elective				Primary, emergent				Redo, elective				Redo, emergent				Artery only	Artery + SVG	SVG only	Others	Unclear
	Cases	30 day mortality		Hospital mortality	Cases	30 day mortality		Hospital mortality	Cases	30 day mortality		Hospital mortality	Cases	30 day mortality		Hospital mortality					
		Hospital	After discharge			Hospital	After discharge			Hospital	After discharge			Hospital	After discharge						
1VD	289	1 (0.3)	1 (0.3)	2 (0.7)	27	0	0	0	5	0	0	0	1	0	0	0	248	48	25	1	0
2VD	858	5 (0.6)	1 (0.1)	6 (0.7)	66	0	0	1 (1.5)	8	1 (12.5)	0	1 (12.5)	0	0	0	0	343	558	28	3	0
3VD	2103	18 (0.9)	1 (0.0)	34 (1.6)	164	5 (3.0)	0	12 (7.3)	10	0	0	0	3	0	0	0	483	1752	32	13	0
LMT	1880	27 (1.4)	3 (0.2)	36 (1.9)	287	13 (4.5)	0	18 (6.3)	14	1 (7.1)	0	2 (14.3)	2	1 (50.0)	0	1 (50.0)	591	1530	54	8	0
no info	77	1 (1.3)	0	1 (1.3)	21	0	0	0	1	0	0	0	4	2 (50.0)	0	3 (75.0)	41	53	8	1	0
Total	5207	52 (1.0)	6 (0.1)	79 (1.5)	565	18 (3.2)	0	31 (5.5)	38	2 (5.3)	0	3 (7.9)	10	3 (30.0)	0	4 (40.0)	1706	3941	147	26	0
Kawasaki	20	0	0	0	0	0	0	0	0	0	0	0	0	0	0	0	14	6	0	0	0
on dialysis	549	20 (3.6)	2 (0.4)	32 (5.8)	69	5 (7.2)	0	7 (10.1)	6	1 (16.7)	0	1 (16.7)	0	0	0	0	141	456	24	3	0
(), % mortality																					
*CABG* coronary artery bypass grafting; *1VD* one-vessel disease; *2VD* two-vessel disease; *3VD* three-vessel disease; *LMT* left main trunk; *SVG* saphenous vein graft																					
LMT includes LMT alone or LMT with other branch diseases																					

(c) Cases of conversion, during surgery, from off-pump CABG to on-pump CABG or on- pump beating-heart CABG (these cases are also included in category (b))																
	Primary, elective				Primary, emergent				Redo, elective				Redo, emergent			
	Cases	30 day mortality		Hospital mortality	Cases	30 day mortality		Hospital mortality	Cases	30 day mortality		Hospital mortality	Cases	30 day mortality		Hospital mortality
		Hospital	After discharge			Hospital	After discharge			Hospital	After discharge			Hospital	After discharge	
Converted to arrest	17	1 (5.9)	0	1 (5.9)	5	0	0	0	0	0	0	0	0	0	0	0
Converted to beating	94	4 (4.3)	1 (1.1)	7 (7.4)	25	2 (8.0)	0	3 (12.0)	1	1 (100.0)	0	1 (100.0)	0	0	0	0
Total	111	5 (4.5)	1 (0.9)	8 (7.2)	30	2 (6.7)	0	3 (10.0)	1	1 (100.0)	0	1 (100.0)	0	0	0	0
On dialysis	17	2 (11.8)	0	3 (17.6)	6	1 (16.7)	0	1 (16.7)	1	1 (100.0)	0	1 (100.0)	0	0	0	0
(), % mortality																
*CABG* coronary artery bypass grafting																

(B) Operation for complications of MI (total; 1114)											
	Chronic				Acute				Concomitant operation		
	Cases	30-day mortality		Hospital mortality	Cases	30-day mortality		Hospital mortality			
		Hospital	After discharge			Hospital	After discharge		CABG	MVP	MVR
Infarctectomy or aneurysmectomy	68	1 (1.5)	0	2 (2.9)	29	10 (34.5)	0	10 (34.5)	45	12	2
VSP closure	69	8 (11.6)	0	12 (17.4)	270	73 (27.0)	0	103 (38.1)	85	3	2
Cardiac rupture	25	4 (16.0)	1(4.0)	6 (24.0)	264	99 (37.5)	0	110 (41.7)	32	2	6
Mitral regurgitation											
1) Papillary muscle rupture	16	1 (6.3)	0	1 (6.3)	59	11 (18.6)	0	18 (30.5)	24	9	66
2) Ischemic	103	4 (3.9)	0	7 (6.8)	38	7 (18.4)	0	10 (26.3)	102	77	64
Others	92	6 (6.5)	1 (1.1)	7 (7.6)	81	20 (24.7)	0	27 (33.3)	71	8	5
Total	373	24 (6.4)	2(0.5)	35 (9.4)	741	220 (29.7)	0	278 (37.5)	359	111	145
(), % mortality											
*MI* myocardial infarction; *CABG* coronary artery bypass grafting; *MVP* mitral valve repair; *MVR* mitral valve replacement; *VSP* ventricular septal perforation											
Acute, within 2 weeks from the onset of myocardial infarction											

(3) Operation for arrhythmia (total; 6728)											
	Cases	30-day mortality		Hospital mortality	Concomitant operation						
					Isolated	Congenital	Valve	IHD	Others	Multiple combination	
		Hospital	After discharge							2 categories	3 categories
Maze	3366	49 (1.5)	1 (0.03)	92 (2.7)	236	178	2760	555	323	638	46
For WPW	9	0	0	0	1	1	7	0	1	1	0
For ventricular tachyarrhythmia	19	2 (10.5)	0	2 (10.5)	3	1	3	6	3	2	0
Others	3334	57 (1.7)	0	113 (3.4)	140	167	2661	661	418	662	49
Total	6728	108 (1.6)	1 (0.01)	207 (3.1)	380	347	5431	1222	745	1303	95
(), % mortality											
*WPW* Wolff–Parkinson–White syndrome; *IHD* ischemic heart disease											
Except for 170 isolated cases, all remaining 5164 cases are doubly allocated, one for this subgroup and the other for the subgroup corresponding to the concomitant operations											

(4) Operation for constrictive pericarditis (total; 166)								
	CPB ( +)				CPB ( −)			
	Cases	30-day mortality		Hospital mortality	Cases	30-day mortality		Hospital mortality
		Hospital	After discharge			Hospital	After discharge	
Total	93	8 (8.6)	0	12 (12.9)	73	7 (9.6)	0	12 (16.4)
(), % mortality								
*CPB* cardiopulmonary bypass								

(5) Cardiac tumor (total; 634)								
	Cases	30-day mortality		Hospital mortality	Concomitant operation			
		Hospital	After discharge		AVR	MVR	CABG	Others
Benign tumor	568	6 (1.1)	0	10 (1.8)	30	10	43	136
(Cardiac myxoma)	378	1 (0.3)	0	4 (1.1)	10	2	15	73
Malignant tumor	66	4 (6.1)	0	7 (10.6)	0	3	2	14
(Primary)	39	2 (5.1)	0	3 (7.7)	0	3	2	5
(), % mortality								
*AVR* aortic valve replacement; *MVR* mitral valve replacement; *CABG* coronary artery bypass grafting								

(6) HOCM and DCM (total; 231)								
	Cases	30-day mortality		Hospital mortality	Concomitant operation			
		Hospital	After discharge		AVR	MVR	MVP	CABG
Myectomy	123	3 (2.4)	0	3 (2.4)	36	19	16	9
Myotomy	6	0	0	0	2	0	1	1
No-resection	96	3 (3.1)	0	5 (5.2)	17	47	49	5
Volume reduction surgery of the left ventricle	6	0	0	1 (16.7)	1	0	2	0
Total	231	6 (2.6)	0	9 (3.9)	56	66	68	15
(), % mortality								
*HOCM* hypertrophic obstructive cardiomyopathy; *DCM* dilated cardiomyopathy; *AVR* aortic valve replacement; *MVR* mitral valve replacement; *MVP* mitral valve repair, *CABG* coronary artery bypass grafting								

(7) Other open-heart operation (total; 1033)				
	Cases	30-day mortality		Hospital mortality
		Hospital	After discharge	
Open-heart operation	439	47 (10.7)	0	63 (14.4)
Non-open-heart operation	594	68 (11.4)	1 (0.2)	93 (15.7)
Total	1033	115 (11.1)	1 (0.1)	156 (15.1)
(), % mortality

**Table 3:** Thoracic aorta (total; 22,597) (1) Dissection (total; 11,438)

Stanford type	Acute								Chronic								Concomitant operation					
		A				B				A				B								
Replaced site	Cases	30-day mortality		Hospital mortality	Cases	30-day mortality		Hospital mortality	Cases	30-day mortality		Hospital mortality	Cases	30-day mortality		Hospital mortality	AVP	AVR	MVP	MVR	CABG	Others
		Hospital	After discharge			Hospital	After discharge			Hospital	After discharge			Hospital	After discharge							
Ascending Ao	2043	150 (7.3)	2 (0.10)	194 (9.5)	2	0	0	0	177	3 (1.7)	0	6 (3.4)	4	1 (25.0)	0	1 (25.0)	41	115	17	10	121	28
Aortic Root	211	32 (15.2)	0	37 (17.5)	2	1 (50.0)	0	1 (50.0)	79	2 (2.5)	0	3 (3.8)	3	0	0	0	20	215	2	5	67	6
Arch	2282	133 (5.8)	2 (0.09)	177 (7.8)	22	2 (9.1)	0	2 (9.1)	368	8 (2.2)	0	13 (3.5)	150	7 (4.7)	0	9 (6.0)	89	157	16	11	111	25
Aortic root + asc. Ao. + Arch	179	15 (8.4)	1 (0.56)	20 (11.2)	1	0	0	0	51	2 (3.9)	0	3 (5.9)	12	0	0	0	26	146	4	1	35	2
Descending Ao	20	1 (5.0)	0	2 (10.0)	19	1 (5.3)	0	1 (5.3)	59	0	0	0	196	5 (2.6)	0	8 (4.1)	0	1	0	0	4	0
Thoracoabdominal	1	0	0	0	9	0	0	0	44	0	1 (2.3)	2 (4.5)	163	5 (3.1)	0	9 (5.5)	0	0	0	0	0	0
Simple TEVAR	91	11 (12.1)	0	16 (17.6)	400	29 (7.3)	1 (0.3)	38 (9.5)	277	6 (2.2)	0	7 (2.5)	1150	12 (1.0)	3 (0.3)	18 (1.6)	0	1	0	0	1	2
Open SG with BR	1440	96 (6.7)	0	123 (8.5)	65	5 (7.7)	0	7 (10.8)	203	7 (3.4)	0	8 (3.9)	277	6 (2.2)	0	9 (3.2)	46	149	6	3	105	11
Open SG without BR	489	49 (10.0)	0	61 (12.5)	25	2 (8.0)	0	2 (8.0)	62	3 (4.8)	0	3 (4.8)	88	4 (4.5)	0	5 (5.7)	13	46	5	0	34	6
Arch TEVAR with BR	15	0	0	0	121	12 (9.9)	1 (0.8)	15 (12.4)	77	0	0	1 (1.3)	421	1 (0.2)	2 (0.5)	2 (0.5)	0	0	0	0	0	0
Thoracoabdominal TEVAR with BR	2	0	0	0	4	0	0	0	11	0	0	0	38	2 (5.3)	0	3 (7.9)	0	0	0	0	0	0
Other	23	11 (47.8)	0	11 (47.8)	14	3 (21.4)	0	4 (28.6)	9	0	0	0	39	2 (5.1)	0	3 (7.7)	0	1	0	0	1	1
Total	6796	498 (7.3)	5 (0.07)	641 (9.4)	684	55 (8.0)	2 (0.3)	70 (10.2)	1417	31 (2.2)	1 (0.1)	46 (3.2)	2541	45 (1.8)	5 (0.2)	67 (2.6)	235	831	50	30	479	81
(), % mortality																						
*Ao* aorta; *AVP* aortic valve repair; *AVR* aortic valve replacement; *MVP* mitral valve repair; *MVR* mitral valve replacement; *CABG* coronary artery bypass grafting; *TEVAR* thoracic endovascular aortic (aneurysm) repair																						
Acute, within 2 weeks from the onset																						

(2) Non-dissection (total; 11,159)														
Replaced site	Unruptured				Ruptured				Concomitant operation					
	Cases	30-day mortality		Hospital mortality	Cases	30-day mortality		Hospital mortality	AVP	AVR	MVP	MVR	CABG	Others
		Hospital	After discharge			Hospital	After discharge							
Ascending Ao	1245	15 (1.2)	1 (0.08)	34 (2.7)	50	7 (14.0)	0	10 (20.0)	29	833	40	51	138	87
Aortic root	1117	27 (2.4)	3 (0.27)	39 (3.5)	68	10 (14.7)	0	16 (23.5)	236	859	71	30	154	62
Arch	1959	32 (1.6)	0	60 (3.1)	89	6 (6.7)	0	10 (11.2)	40	587	29	24	230	69
Aortic root + asc. Ao. + Arch	232	4 (1.7)	0	6 (2.6)	7	0	0	0	39	175	10	3	24	3
Descending Ao	286	13 (4.5)	0	26 (9.1)	27	4 (14.8)	0	7 (25.9)	1	7	0	0	15	1
Thoracoabdominal	353	20 (5.7)	1 (0.28)	29 (8.2)	33	6 (18.2)	0	12 (36.4)	0	0	0	0	1	0
Simple TEVAR	2283	27 (1.2)	3 (0.13)	56 (2.5)	365	38 (10.4)	3 (0.82)	60 (16.4)	0	2	0	0	2	6
Open SG with BR	1088	30 (2.8)	0	61 (5.6)	74	7 (9.5)	0	12 (16.2)	13	118	7	6	170	16
Open SG without BR	404	9 (2.2)	0	16 (4.0)	29	1 (3.4)	0	3 (10.3)	6	71	9	5	44	6
Arch TEVAR with BR	1121	20 (1.8)	1	38 (3.4)	66	11 (16.7)	0	18 (27.3)	0	0	0	0	4	1
Thoracoabdominal TEVAR with BR	103	8 (7.8)	0	9 (8.7)	4	1 (25.0)	0	1 (25.0)	0	0	0	0	0	0
Other	140	5 (3.6)	0	10 (7.1)	16	3 (18.8)	0	4 (25.0)	1	10	2	1	4	1
Total	10,331	210 (2.0)	9 (0.09)	384 (3.7)	828	94 (11.4)	3 (0.36)	153 (18.5)	365	2662	168	120	786	252
(), % mortality														
*Ao* aorta; *AVP* aortic valve repair; *AVR* aortic valve replacement; *MVP* mitral valve repair; *MVR* mitral valve replacement; *CABG* coronary artery bypass grafting; *TEVAR* thoracic endovascular aortic(aneurysm) repair

**Table 4:** Pulmonary thromboembolism (total; 186)

	Cases	30-day mortality		Hospital mortality
		Hospital	After discharge	
Acute	119	12 (10.1)	0	14 (11.8)
Chronic	67	0	0	1 (1.5)
Total	186	12 (6.5)	0	15 (8.1)

(), % mortality

**Table 5:** Implantation of VAD (total; 120)

	Cases	30-day mortality		Hospital mortality
		Hospital	After discharge	
Implantation of VAD	120	1 (0.8)	0	9 (7.5)

(), % mortality

*VAD* ventricular assist devise

**Table 6:** Heart Transplantation (total; 79)

	Cases	Hospital mortality
Heart transplantation	79	5 (6.3)
Heart and lung transplantation	0	0
Total	79	5 (6.3)

(), % mortality

Among the 7580 procedures for congenital heart disease conducted in 2022, 5789 were open-heart surgeries, with an overall hospital mortality rate of 1.7% (Table 1). The number of surgeries for neonates and infants in 2022 significantly decreased compared to that in 2012 (3645 vs 4927); on the other hands, hospital mortality did not significantly differ compared to those in 2012 (7.2% vs 5.6% for neonates and 2.5% vs 2.4% for infants) despite the increasing ratio of surgeries for severe cases. In 2022, atrial septal defect (1193 cases) and ventricular septal defect (1189 cases) were the most common diseases as previously reported, with patients aged ≥ 18 years accounting for 39% of atrial septal defect and ventricular septal defect surgeries.

Hospital mortality of open heart surgeries for complex congenital heart disease within the past 10 years was as follows (2012 [6], 2017 [7], and 2022): complete atrioventricular septal defect (3.2%, 2.7%, and 2.9%); tetralogy of Fallot (1.1%, 0.9%, and 0.8%); transposition of the great arteries with the intact septum (2.6%, 4.5%, and 3.8%), ventricular septal defect (3.2%, 1.5%, and 7.3%), single ventricle (5.5%, 2.2%, and 2.8%); and hypoplastic left heart syndrome (10.2%, 8.8%, and 8.2%). Currently, right heart bypass surgery has been commonly performed (225 bidirectional Glenn procedures, excluding 22 Damus–Kaye–Stansel procedures, and 289 Fontan type procedures, including total cavopulmonary connection) with acceptable hospital mortality rates (0.9% and 0.0%). The Norwood type I procedure was performed in 111 cases, with a relatively low hospital mortality rate (13.5%) (Table 1).

Valvular heart disease procedures, excluding transcatheter procedures, were performed less than that in the previous year. Isolated aortic valve replacement/repair with/without coronary artery bypass grafting (CABG) (*n* = 7834) was 4.7% fewer than that in the previous year (*n* = 8206) and 36.5% fewer than that 5 years ago (*n* = 10,690 in 2017), as opposed to the rapid increase of transcatheter aortic valve replacement (*n* = 12,202 and 13,534 in 2021 and 2022). Isolated mitral valve replacement/repairs with/without CABG (*n* = 4708) was 6.6% more than that in the previous year (*n* = 4415) and 0.4% more than that 5 years ago (*n* = 4687 in 2017). Aortic and mitral valve replacement with bioprosthesis were performed in 8126 and 2313 cases, respectively. The rate at which bioprosthesis was used had dramatically increased from 30% in the early 2000s [8, 9] to 87.2% and 72.7% in 2022 for aortic and mitral positions, respectively. Additionally, CABG was performed concurrently in 15.9% of all valvular procedures (18.2% in 2012 [6] and 17.8% in 2017 [7]). Valve repair was common in mitral and tricuspid valve positions (5768 and 3558 cases, respectively) but less common in aortic valve positions (116 patients, only 1.2% of all aortic valve procedures). Mitral valve repair accounted for 65.2% of all mitral valve procedures. Hospital mortality rates for isolated valve replacement for aortic and mitral positions were 2.5% and 7.3%, respectively, but only 1.1% for mitral valve repair. Moreover, hospital mortality rates for redo isolated valve surgery for the aortic and mitral positions were 4.8% and 4.9%, respectively. Finally, overall hospital mortality rates did not significantly improve over the past 10 years (3.2% in 2012 [6], 3.6% in 2017 [7], and 2.8% in 2022) (Table 2).

Isolated CABG had been performed in 10,226 cases, accounting for only 66.1% of the procedures performed 10 years ago (*n* = 15,462 in 2012) [6]. Of the aforementioned cases, 5820 (56.9%) underwent off-pump CABG, with a success rate of 97.6%. The percentage of planned off-pump CABG in 2022 was similar to that in 2021. Hospital mortality associated with primary elective CABG procedures among 8923 cases accounted for 1.9%, which is slightly higher than that in 2012 (1.1%) [6]. Hospital mortality for primary emergency CABG among 1207 cases remained high (9.4%). The percentage of conversion from off-pump to on-pump CABG or on-pump beating-heart CABG was 2.1% among the primary elective CABG cases, with a hospital mortality rate of 7.2%. Patients with end-stage renal failure on dialysis had higher hospital mortality rates than overall mortality, regardless of surgical procedure (on-pump arrest, on-pump beating, and off-pump). This study excluded concomitant CABGs alongside other major procedures under the ischemic heart disease category but rather under other categories, such as valvular heart disease and thoracic aortic aneurysm. Accordingly, the overall number of CABGs in 2022, including concomitant CABG with other major procedures, was 14,656 (Table 2).

Arrhythmia management was primarily performed as concomitant procedures in 6728 cases, with a hospital mortality rate of 3.1%. Pacemaker and implantable cardioverter-defibrillator implantation were not included in this category (Table 2).

In 2022, 22,597 procedures for thoracic and thoracoabdominal aortic diseases were performed, among which aortic dissection and non-dissection accounted for 11,438 and 11,159, respectively. The number of surgeries for aortic dissection this year was 1.7% higher than that in the preceding year (*n* = 11,247 in 2021). Hospital mortality rates for the 6,796 Stanford type A acute aortic dissections remained high (9.4%). The number of procedures for non- aortic dissections decreased by 5.2%, with a hospital mortality rate of 4.8% for all aneurysms and 3.7% and 18.5% for unruptured and ruptured aneurysms, respectively. Thoracic endovascular aortic repair (TEVAR) has been performed for aortic diseases at an increasing rate [2–4]. Stent graft placement was performed in 5256 patients with aortic dissection, including 2607 TEVARs and 2649 open stent graftings. Moreover, 1609 and 365 cases underwent TEVAR and open stent grafting for type B chronic aortic dissection, accounting for 61.7% and 13.8% of the total number of cases, respectively. Hospital mortality rates associated with simple TEVAR for type B aortic dissection were 9.5% and 1.6% for acute and chronic cases, respectively. Stent graft placement was performed in 5,537 patients with non-dissected aortic aneurysms, among which 3942 were TEVARs (a 5.1% decrease compared to that in 2021, *n* = 4143) and 1595 were open stent graftings (an 11.8% decrease compared to that in 2021, *n* = 1783). Hospital mortality rates were 2.9% and 18.2% for TEVARs and 5.2% and 14.6% for open stenting in unruptured and ruptured aneurysms, respectively (Table 3).

### (B) General thoracic surgery

**Fig. 2:**
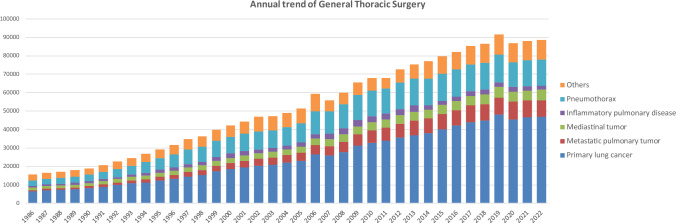
Annual trend of general thoracic surgery

The 2022 survey of general thoracic surgeries comprised 707 surgical units, with bulk data submitted via a web-based collection system established by the NCD [4]. General thoracic surgery departments reported 88,697 procedures in 2022 (Table 7), which is 2.1 times more than that in 2000 and 3390 more procedures than that in 2017 [7] (Fig. 2). It increased compared to that in 2020 (the first year of COVID-19 pandemic: 86,813) [3] by 2.2%. However it still decreased by 3.3% compared to that of 2019 (before COVID-19 pandemic: 91,626) [2], mostly because of the protraction of COVID-19 pandemic, despite the steadily increase up to 2019.

**Table 7:** Total cases of general thoracic surgery during 2022

	Cases	%
Benign pulmonary tumor	2385	2.7
Primary lung cancer	46,888	52.9
Other primary malignant pulmonary tumor	408	0.5
Metastatic pulmonary tumor	9055	10.2
Tracheal tumor	99	0.1
Pleural tumor including mesothelioma	588	0.7
Chest wall tumor	577	0.7
Mediastinal tumor	5652	6.4
Thymectomy for MG without thymoma	128	0.1
Inflammatory pulmonary disease	2062	2.3
Empyema	3459	3.9
Bullous disease excluding pneumothorax	267	0.3
Pneumothorax	14,459	16.3
Chest wall deformity	298	0.3
Diaphragmatic hernia including traumatic	29	0.0
Chest trauma excluding diaphragmatic hernia	508	0.6
Lung transplantation	109	0.1
Others	1726	1.9
Total	88,697	100.0

In 2022, 46,888 procedures for primary lung cancer had been performed which increased by 0.6% compared to that of 2021 (46,624) [4], but still decreased by 2.5% compared to that of 2019 (48,052) [2], similarly to the total number of surgeries in general thoracic surgery. The number of procedures in 2022 was 2.5 times higher than that in 2000, with lung cancer procedures accounting for 53% of all general thoracic surgeries.

Information about the number of video-assisted thoracoscopic surgery (VATS), which is defined as surgical procedures using a skin incision less than 8 cm including a mini-thoracotomy (hybrid) approach, have been available since the 2015 annual report. Tables 8, 9, 11, 14, 15, 16, 18, 19, 22, 24, and 25 present the number of VATS procedures for benign pulmonary tumors, primary lung cancer, metastatic pulmonary tumor, chest wall tumor, mediastinal tumor, thymectomy for myasthenia gravis, inflammatory pulmonary disease, empyema, descending necrotizing mediastinitis, bullous diseases, pneumothorax, diaphragmatic hernia, chest trauma and other respiratory surgeries in 2021, respectively.

**Table 8:** Benign pulmonary tumor

	Cases	30-day mortality		Hospital mortality	By VATS
		Hospital	After discharge		
1. Benign pulmonary tumor					
Hamartoma	495	0	0	0	479
Sclerosing hemangioma	106	0	0	0	99
Papilloma	27	0	0	0	27
Mucous gland adenoma bronchial	24	0	0	0	23
Fibroma	133	0	0	0	122
Lipoma	9	0	0	0	8
Neurogenic tumor	11	0	0	0	11
Clear cell tumor	1	0	0	0	1
Leiomyoma	15	0	0	0	15
Chondroma	4	0	0	0	4
Inflammatory myofibroblastic tumor	2	0	0	0	2
Pseudolymphoma	17	0	0	0	16
Histiocytosis	10	0	0	0	10
Teratoma	5	0	0	0	5
Others	1526	1 (0.1)	0	2 (0.1)	1432
Total	2385	1 (0.04)	0	2 (0.08)	2254

(), Mortality %

**Table 9:** Primary malignant pulmonary tumor

	Cases	30-Day mortality		Hospital mortality	VATS	Robotic surgery
		Hospital	After discharge			
2. Primary malignant pulmonary tumor	47,296	98 (0.2)	62 (0.1)	183 (0.4)	34,539	5497
Lung cancer	46,888	98 (0.2)	61 (0.1)	182 (0.4)	34,539	5497
Histological classification						
Adenocarcinoma	33,272	42 (0.1)	29 (0.09)	70 (0.2)		
Squamous cell carcinoma	8029	36 (0.4)	20 (0.2)	76 (0.9)		
Large cell carcinoma	335	2 (0.6)	0	4 (1.2)		
LCNEC	556	3 (0.5)	3 (0.5)	5 (0.9)		
Small cell carcinoma	789	3 (0.4)	2 (0.3)	5 (0.6)		
Adenosquamous carcinoma	550	6 (1.1)	1 (0.2)	7 (1.3)		
Carcinoma with pleomorphic, sarcomatoid or sarcomatous elements	543	1 (0.2)	3 (0.6)	6 (1.1)		
Carcinoid	248	0	0	0		
Carcinomas of salivary-gland type	43	0	0	0		
Unclassified	33	0	0	0		
Multiple lung cancer	2114	3 (0.1)	3 (0.1)	5 (0.2)		
Others	336	2 (0.6)	0	4 (1.2)		
Operative procedure						
Wedge resection	8941	14 (0.2)	10 (0.1)	27 (0.3)	8332	24
Segmental excision	7999	5 (0.1)	5 (0.06)	10 (0.1)	6295	1008
(Sleeve segmental excision)	10	0	0	0	3	1
Lobectomy	29,511	69 (0.2)	45 (0.15)	131 (0.4)	19,723	4461
(Sleeve lobectomy)	310	4 (1.3)	2 (0.6)	8 (2.6)	22	8
Pneumonectomy	174	6 (3.4)	1 (0.6)	8 (4.6)	22	3
(Sleeve pneumonectomy)	3	0	0	0	0	0
Other bronchoplasty	34	1 (2.9)	0	1 (2.9)	2	0
Pleuropneumonectomy	0	0	0	0	0	0
Others	192	3 (1.6)	0	5 (2.6)	132	0
Multiple incision for multiple lung cancer	37	0	0	0	33	1
Sarcoma	32	0	0	1 (3.1)		
AAH	124	0	0	0		
Lymphoma	185	0	1 (0.5)	0		
Others	67	0	0	0		

(), Mortality %

A total of 2385 procedures for benign pulmonary tumors had been conducted in 2022 (Table 8). Hamartomas were the most frequent benign pulmonary tumors diagnosed, with 2254 patients (95%) undergoing VATS.

Tables 9 and 10 show additional information on primary malignant pulmonary tumors. Accordingly, the most frequently diagnosed lung cancer subtype was adenocarcinoma (71% of all lung cancers), followed by squamous cell carcinoma (17%). Sublobar resection was performed in 16,940 lung cancer cases (36% of all cases) and lobectomy in 29,511 cases (63% of all cases). Sleeve lobectomy was performed in 310 cases (0.7% of all cases), while pneumonectomy was required in 174 cases (0.4% of all cases). VATS lobectomy was performed in 19,723 cases of lung cancer (67% of all lobectomy cases). RATS lobectomy was performed in 4461 cases of lung cancer (15% of all lobectomy cases). Patients aged ≥ 80 years who underwent lung cancer surgery accounted for 7404 (16%). Among those who died within 30 days postoperatively, 98 and 62 died before and after hospital discharge, respectively. Overall, 160 patients died within 30 days postoperatively (30-day mortality rate, 0.3%), while 183 died before discharge (hospital mortality rate, 0.4%). Moreover, 30-day mortality rates according to the procedure were 0.1%, 0.2%, and 3% for segmentectomy, lobectomy, and pneumonectomy, respectively. Interstitial pneumonia had been the leading cause of death after lung cancer surgery, followed by pneumonia, cardiovascular events, respiratory failure, and brain infarction or bleeding.

**Table 10:** Details of lung cancer operations

TNM	
c-Stage	Cases
0	2212
IA1	9102
IA2	13,894
IA3	8069
IB	4902
IIA	1595
IIB	3653
IIIA	2442
IIIB	409
IIIC	16
IVA	370
IVB	84
NA	103
Total	46,851

Sex	Cases
Male	27,930
Female	18,921
NA	0
Total	46,851

Cause of death	Cases
Cardiovascular	37
Pneumonia	39
Pyothorax	8
Bronchopleural fistula	10
Respiratory failure	23
Pulmonary embolism	6
Interstitial pneumonia	100
Brain infarction or bleeding	22
Others	134
Unknown	29
Total	408

p-Stage	Cases
0(pCR)	3236
IA1	9549
IA2	11,076
IA3	5287
IB	6580
IIA	1313
IIB	4253
IIIA	3613
IIIB	717
IIIC	12
IVA	856
IVB	84
NA	272
Total	46,848

Age (y)	Cases
< 20	18
20–29	45
30–39	230
40–49	1138
50–59	3836
60–69	10,658
70–79	23,522
80–89	7273
≥ 90	131
NA	0
Total	46,851

The procedures for metastatic pulmonary tumors performed in 2022 (9055) was similar to that in 2021 (9047) [4] (Table 11). Among such procedures, the most frequent primary tumor was colorectal cancer (47% of all cases).

**Table 11:** Metastatic pulmonary tumor

	Cases	30-day mortality		Hospital mortality	VATS	Robotic surgery
		Hospital	After discharge			
3. Metastatic pulmonary tumor	9055	5 (0.1)	6 (0.07)	11 (0.12)	8329	361
Colorectal	4243	1 (0.02)	1 (0.02)	2 (0.05)	3913	190
Hepatobiliary/pancreatic	507	0	0	1 (0.20)	485	23
Uterine	502	0	0	0	458	21
Mammary	575	0	0	0	554	21
Ovarian	67	0	0	0	63	5
Testicular	46	0	0	0	42	2
Renal	763	2 (0.3)	0	2 (0.26)	704	34
Skeletal	111	0	0	0	98	1
Soft tissue	287	0	1 (0.3)	0	255	12
Otorhinolaryngological	460	0	1 (0.2)	0	423	16
Pulmonary	484	1 (0.2)	1 (0.2)	2 (0.41)	384	8
Others	1010	1 (0.1)	2 (0.2)	4 (0.40)	950	28

(), Mortality %

A total of 99 procedures for tracheal tumors, including 40, 33, and 26 cases of primary malignant, metastatic, and benign tracheal tumors, respectively, were performed in 2022. Further, 15 patients underwent sleeve resection and reconstruction (Table 12).

**Table 12:** Tracheal tumor

	Cases	30-day mortality		Hospital mortality
		Hospital	After discharge	
4. Tracheal tumor	99	0	0	4 (4.0)
A. Primary malignant tumor				
Histological classification				
Squamous cell carcinoma	11	0	0	0
Adenoid cystic carcinoma	19	0	0	0
Mucoepidermoid carcinoma	5	0	0	0
Others	5	0	0	0
Total	40	0	0	0
B. Metastatic/invasive malignant tumor e.g. invasion of thyroid cancer				
	33	0	0	4 (12.1)
C. Benign tracheal tumor				
Histological classification				
Papilloma	6	0	0	0
Adenoma	1	0	0	0
Neurofibroma	1	0	0	0
Chondroma	0	0	0	0
Leiomyoma	0	0	0	0
Others	18	0	0	0
Histology unknown	0	0	0	0
Total	26	0	0	0
Operation				
Sleeve resection with reconstruction	15	0	0	0
Wedge with simple closure	2	0	0	0
Wedge with patch closure	1	0	0	0
Total laryngectomy with tracheostomy	0	0	0	0
Others	1	0	0	0
Unknown	0	0	0	0
Total	19	0	0	0

(), Mortality %

Overall, 588 pleural tumors had been diagnosed in 2022 (Table 13), with diffuse malignant pleural mesothelioma as the most frequent histologic diagnosis. Total pleurectomy was performed in 144 cases and extrapleural pneumonectomy in 21 cases. The 30-day mortality rate was 1.4% and 9.5% after total pleurectomy and extrapleural pneumonectomy, respectively.

**Table 13:** Tumor of pleural origin

5. Tumor of pleural origin				
Histological classification	Cases	30-day mortality		Hospital mortality
		Hospital	After discharge	
Solitary fibrous tumor	113	0	0	0
Diffuse malignant pleural mesothelioma	219	5 (2.3)	0	8 (3.7)
Localized malignant pleural mesothelioma	26	0	0	0
Others	230	1 (0.4)	0	3 (1.3)
Total	588	6 (1.0)	0	11 (1.9)

Operative procedure	Cases	30-day mortality		Hospital mortality
		Hospital	After discharge	
Extrapleural pneumonectomy	21	2 (9.5)	0	2 (9.5)
Total pleurectomy	144	2 (1.4)	0	4 (2.8)
Others	54	1 (1.9)	0	2 (3.7)
Total	219	5 (2.3)	0	8 (3.7)

(), Mortality %

Overall, 577 chest wall tumor resections had been performed in 2022, including 109, 152, and 316 cases of primary malignant, metastatic, and benign tumors, respectively (Table 14).

**Table 14:** Chest wall tumor

	Cases	30-day mortality		Hospital mortality	VATS
		Hospital	After discharge		
6. Chest wall tumor					
Primary malignant tumor	109	1 (0.9)	1 (0.9)	2 (1.8)	35
Metastatic malignant tumor	152	0	0	2 (1.3)	42
Benign tumor	316	0	0	0	242
Total	577	1 (0.2)	1 (0.2)	4 (0.7)	319

(), Mortality %

In 2022, 5652 mediastinal tumors were resected, which slightly increased by 1.1% that in 2021 (5590) (Table 15) [4]. Thymic epithelial tumors, including 2256 thymomas, 370 thymic carcinomas, and 57 thymic carcinoids, were the most frequently diagnosed mediastinal tumor subtype in 2022.

**Table 15:** Mediastinal tumor

	Cases	30-day mortality		Hospital mortality	By VATS	Robotic surgery
		Hospital	After discharge			
7. Mediastinal tumor	5652	8 (0.14)	1 (0.02)	9 (0.2)	4261	1560
Thymoma	2256	3 (0.1)	0	3 (0.1)	1534	629
Thymic cancer	370	2 (0.5)	0	2 (0.5)	206	79
Thymus carcinoid	57	0	0	1 (1.8)	35	11
Germ cell tumor	82	0	0	0	41	13
Benign	53	0	0	0	35	11
Malignant	29	0	0	0	6	2
Neurogenic tumor	467	0	0	0	434	130
Congenital cyst	1203	1 (0.1)	0	1 (0.1)	1133	435
Goiter	80	0	0	0	33	8
Lymphatic tumor	175	1 (0.6)	0	1 (0.6)	132	36
Excision of pleural recurrence of thymoma	44	0	0	0	26	0
Thymolipoma	17	0	0	0	13	7
Others	901	1 (0.1)	1 (0.1)	1 (0.1)	674	212

(), Mortality %

A total of 499 patients underwent thymectomy for myasthenia gravis (Table 16), among which 371 procedures were associated with thymoma in 2022.

**Table 16:** Thymectomy for myasthenia gravis

	Cases	30-day mortality		Hospital mortality	By VATS	Robotic surgery
		Hospital	After discharge			
8. Thymectomy for myasthenia gravis	499	3 (0.6)	0	3 (0.6)	319	146
With thymoma	371	3 (0.8)	0	3 (0.8)	227	104

(), Mortality %

Overall, 22,808 patients underwent procedures for non-neoplastic disease in 2022. Accordingly, 2062 patients underwent lung resection for inflammatory lung diseases (Tables 17, 18), among which 365 and 273 patients were associated with mycobacterial and fungal infections, respectively. Procedures for inflammatory pseudotumor were performed in 916 cases (44%).

**Table 17:** Operations for non-neoplastic diseases:A + B + C + D + E + F + G + H + I

	Cases	30-day mortality		Hospital mortality
		Hospital	After discharge	
9. Operations for non-neoplastic diseases	22,808	260 (1.1)	33 (0.1)	506 (2.2)

**Table 18:** A. Inflammatory pulmonary disease

	Cases	30-day mortality		Hospital mortality	VATS
		Hospital	After discharge		
A. Inflammatory pulmonary disease	2062	8 (0.4)	1 (0.0)	16 (0.8)	1831
Tuberculous infection	32	0	0	0	25
Mycobacterial infection	365	1 (0.3)	0	1 (0.3)	321
Fungal infection	273	3 (1.1)	1 (0.4)	6 (2.2)	193
Bronchiectasis	44	1 (2.3)	0	1 (2.3)	31
Tuberculous nodule	46	0	0	0	45
Inflammatory pseudotumor	916	2 (0.2)	0	4 (0.4)	884
Interpulmonary lymph node	63	1 (1.6)	0	1 (1.6)	62
Others	323	0	0	3 (0.9)	270

(), Mortality %

A total of 3459 procedures were performed for empyema (Table 19), among which 2877 (80%) were acute and 582 (20%) were chronic. Further, pleural fistulas developed in 568 and 256 patients with acute and chronic empyema, respectively. The hospital mortality rate was 13% among patients with acute empyema with fistula.

**Table 19:** B. Empyema

	Cases	30-day mortality		Hospital mortality	By VATS
		Hospital	After discharge		
Acute empyema	2877	78 (2.7)	7 (0.2)	157 (5.5)	2335
With fistula	568	31 (5.5)	3 (0.5)	74 (13.0)	287
Without fistula	2290	46 (2.0)	4 (0.2)	81 (3.5)	2030
Unknown	19	1 (5.3)	0	2 (10.5)	18
Chronic empyema	582	18 (3.1)	2 (0.3)	48 (8.2)	311
With fistula	256	7 (2.7)	0	26 (10.2)	80
Without fistula	281	7 (2.5)	2 (0.7)	13 (4.6)	205
Unknown	45	4 (8.9)	0	9 (20.0)	26
Total	3459	96 (2.8)	9 (0.3)	205 (5.9)	2646

(), Mortality %

Further, 128 operations were performed for descending necrotizing mediastinitis (Table 20), with a hospital mortality rate of 10%.

**Table 20:** C. Descending necrotizing mediastinitis

	Cases	30-day mortality		Hospital mortality	VATS
		Hospital	After discharge		
C. Descending necrotizing mediastinitis	128	9 (7.0)	0	13 (10.2)	95

(), Mortality %

A total of 267 procedures were conducted for bullous diseases (Table 21), while only 15 patients underwent lung volume reduction surgery.

**Table 21:** D. Bullous diseases

	Cases	30-day mortality		Hospital mortality	VATS
		Hospital	After discharge		
D. Bullous diseases	267	0	0	0	252
Emphysematous bulla	195	0	0	0	186
Bronchogenic cyst	18	0	0	0	17
Emphysema with LVRS	15	0	0	0	13
Others	39	0	0	0	36

(), Mortality %

*LVRS* lung volume reduction surgery

A total of 14,459 procedures were performed for pneumothorax (Table 22). Among the 10,261 procedures for spontaneous pneumothorax, 2377 (23%) were bullectomies alone, while 7223 (70%) required additional procedures, such as coverage with artificial material, as well as parietal pleurectomy. A total of 4198 procedures for secondary pneumothorax were performed, with chronic obstructive pulmonary disease (COPD) being the most prevalent associated disease (2943 cases, 70%). The hospital mortality rate for secondary pneumothorax associated with COPD was 2.7%.

**Table 22:** E. Pneumothorax

Cases	30-Day mortality		Hospital mortality	VATS
	Hospital	After discharge		
14,459	98 (0.7)	19 (0.1)	174 (1.2)	14,009

Spontaneous pneumothorax					
Operative procedure	Cases	30-day mortality		Hospital mortality	VATS
		Hospital	After discharge		
Bullectomy	2377	3 (0.1)	1 (0.0)	3 (0.1)	2318
Bullectomy with additional procedure	7223	7 (0.1)	5 (0.07)	11 (0.2)	7138
Coverage with artificial material	7012	7 (0.1)	5 (0.07)	10 (0.1)	6935
Parietal pleurectomy	45	0	0	0	41
Coverage and parietal pleurectomy	67	0	0	0	66
Others	99	0	0	1 (1.0)	96
Others	659	3 (0.5)	0	12 (1.8)	605
Unknown	2	0	0	0	1
Total	10,261	13 (0.1)	6 (0.1)	26 (0.3)	10,062

Secondary pneumothorax					
Associated disease	Cases	30-day mortality		Hospital mortality	VATS
		Hospital	After discharge		
COPD	2943	45 (1.5)	9 (0.3)	80 (2.7)	2797
Tumorous disease	158	8 (5.1)	3 (1.9)	13 (8.2)	148
Catamenial	194	0	0	0	189
LAM	32	0	0	0	32
Others (excluding pneumothorax by trauma)	871	32 (3.7)	1 (0.1)	55 (6.3)	781
Unknown	0	0	0	0	0

Operative procedure	Cases	30 day mortality		Hospital mortality	VATS
		Hospital	After discharge		
Bullectomy	721	12 (1.7)	1 (0.1)	17 (2.4)	704
Bullectomy with additional procedure	2450	37 (1.5)	8 (0.3)	59 (2.4)	2370
Coverage with artificial material	2371	32 (1.3)	7 (0.3)	53 (2.2)	2298
Parietal pleurectomy	2	0	0	0	2
Coverage and parietal pleurectomy	25	1 (4.0)	0	2 (8.0)	21
Others	52	4 (7.7)	1 (1.9)	4 (7.7)	49
Others	1019	36 (3.5)	4 (0.4)	71 (7.0)	868
Unknown	8	0	0	1 (12.5)	5
Total	4198	85 (2.0)	13 (0.3)	148 (3.5)	3947

(), Mortality %

The 2022 survey reported 298 procedures for chest wall deformity (Table 23). However, this may have been underestimated because the Nuss procedure for pectus excavatum was more likely performed in pediatric surgery centers not associated with the Japanese Association for Thoracic Surgery.

**Table 23:** F. Chest wall deformity

	Cases	30-day mortality		Hospital mortality
		Hospital	After discharge	
F. Chest wall deformity	298	0	0	0
Funnel chest	291	0	0	0
Others	7	0	0	0

(), Mortality %

Surgical treatment for diaphragmatic hernia was performed in 29 patients (Table 24). This may have been underestimated because procedures may have been classified as gastrointestinal surgery.

**Table 24:** G. Diaphragmatic hernia

	Cases	30-day mortality		Hospital mortality	VATS
		Hospital	After discharge		
G. Diaphragmatic hernia	29	0	0	1 (3.4)	14
Congenital	9	0	0	1 (11.1)	2
Traumatic	6	0	0	0	1
Others	14	0	0	0	11

(), Mortality %

The survey reported 508 procedures for chest trauma, excluding iatrogenic injuries (Table 25), with a hospital mortality rate of 5.3%.

**Table 25:** H. Chest trauma

	Cases	30-Day mortality		Hospital mortality	VATS
		Hospital	After discharge		
H. Chest trauma	508	14 (2.8)	1 (0.2)	27 (5.3)	307

(), Mortality %

Table 26 summarizes the procedures for other diseases, including 94 and 107 cases of arteriovenous malformation and pulmonary sequestration, respectively.

**Table 26:** I. Other respiratory surgery

	Cases	30-day mortality		Hospital mortality	VATS
		Hospital	After discharge		
I. Other respiratory surgery	1598	35 (2.2)	3 (0.2)	70 (4.4)	1154
Arteriovenous malformation	94	1 (1.1)	0	1 (1.1)	88
Pulmonary sequestration	107	0	0	0	90
Postoperative bleeding air leakage	468	12 (2.6)	1 (0.2)	31 (6.6)	299
Chylothorax	55	1 (1.8)	0	1 (1.8)	51
Others	874	21 (2.4)	2 (0.2)	37 (4.2)	626

(), Mortality %

A total of 109 lung transplantations were performed in 2022 (Table 27), among which 94 and 15 were from brain-dead and living-related donors, respectively. 30-day mortality for total lung transplantation was 0.9% (1/109).

**Table 27:** Lung transplantation

	Cases	30-day mortality		Hospital mortality
		Hospital	After discharge	
Single lung transplantation from brain-dead donor	48	1 (2.1)	0	1 (2.1)
Bilateral lung transplantation from brain-dead donor	46	0	0	1 (2.2)
Lung transplantation from living donor	15	0	0	0
Total lung transplantation	109	1 (0.9)	0	2 (1.8)
Donor of living donor lung transplantation	27	0	0	0
Donor of brain-dead donor lung transplantation	80			

(), Mortality %

In 2022, the number of VATS procedures increased by 1.7% from 76,073 to 77,405 compared to that of 2021. The population of VATS procedures in all procedures 87% in 2022 was similar to that in 2021 (88%) (Table 28).

**Table 28:** Video-assisted thoracic surgery

	Cases	30-day mortality		Hospital mortality
		Hospital	After discharge	
11. Video-assisted thoracic surgery	77,405	237 (0.3)	78 (0.1)	442 (0.6)

(), Mortality % (including thoracic sympathectomy 172)

A total of 577 tracheobronchoplasty procedures were performed in 2022, including 314 sleeve lobectomies, 15 carinal reconstructions and 4 sleeve pneumonectomies (Table 29). 30-day mortality for sleeve lobectomy, carinal reconstruction and sleeve pneumonectomy were 1.9, 0 and 0% respectively.

**Table 29:** Tracheobronchoplasty

	Cases	30-day mortality		Hospital mortality
		Hospital	After discharge	
12. Tracheobronchoplasty	577	10 (1.7)	2 (0.3)	20 (3.5)
Trachea	41	1 (2.4)	0	2 (4.9)
Sleeve resection with reconstruction	21	0	0	0
Wedge with simple closure	7	0	0	0
Wedge with patch closure	1	0	0	0
Total laryngectomy with tracheostomy	0	0	0	0
Others	12	1 (8.3)	0	2 (16.7)
Carinal reconstruction	15	0	0	0
Sleeve pneumonectomy	4	0	0	0
Sleeve lobectomy	314	4 (1.3)	2 (0.6)	8 (2.5)
Sleeve segmental excision	10	0	0	0
Bronchoplasty without lung resection	18	0	0	0
Others	175	5 (2.9)	0	10 (5.7)

(), Mortality %

A total of 343 pediatric surgery was performed in 2022 with hospital mortality of 2% (Table 30).

**Table 30:** Pediatric surgery

	Cases	30-day mortality		Hospital mortality
		Hospital	After discharge	
13. Pediatric surgery	343	7 (2.0)	0	7 (2.0)

(), Mortality %

Overall, 1186 combined resection of neighboring organ(s) had been performed for primary lung cancer and mediastinal tumor in 2022. The combines resection for primary lung cancer includes 243, 94, 73, 48, 18, 13 and 6 cases of chest wall, pulmonary artery, pericardium, diaphragm, superior vena cava, left atrium and aorta resections, respectively. The combines resection for mediastinal tumor includes 521, 354, 101, 41, 41 and 11 cases of lung, pericardium, brachiocephalic vein, superior vena cava, diaphragm and chest wall resections, respectively (Table 31).

**Table 31:** Combined resection of neighboring organ(s)

	Cases	30-day mortality		Hospital mortality
		Hospital	After discharge	
14. Combined resection of neighboring organ(s)	1186	8 (0.7)	3 (0.3)	12 (1.0)

Organ resected	Cases	30-day mortality		Hospital mortality
		Hospital	After discharge	
A. Primary lung cancer				
Aorta	6	1 (16.7)	0	1 (16.7)
Superior vena cava	18	1 (5.6)	0	1 (5.6)
Brachiocephalic vein	2	0	0	0
Pericardium	73	1 (1.4)	0	1 (1.4)
Pulmonary artery	94	0	3 (3.2)	1 (1.1)
Left atrium	13	1 (7.7)	0	1 (7.7)
Diaphragm	48	0	0	0
Chest wall (including ribs)	243	1 (0.4)	0	4 (1.6)
Vertebra	4	0	0	0
Esophagus	3	0	0	0
Total	504	5 (1.0)	3 (0.6)	9 (1.8)
B. Mediastinal tumor				
Aorta	5	0	0	0
Superior vena cava	41	0	0	0
Brachiocephalic vein	101	1 (1.0)	0	1 (1.0)
Pericardium	354	2 (0.6)	0	2 (0.6)
Pulmonary artery	1	0	0	0
Left atrium	0	0	0	0
Diaphragm	41	0	0	0
Chest wall (including ribs)	11	0	0	0
Vertebra	5	0	0	0
Esophagus	4	0	0	0
Lung	521	3 (0.6)	0	3 (0.6)
Total	1084	6 (0.6)	0	6 (0.6)

(), Mortality %

A total of 610 operations of lung cancer invading the chest wall of apex had been performed in 2022 with hospital mortality of 0.3% (Table 32). A total of 5166 diagnostic procedures were performed in 2022 (Table 33).

**Table 32:** Operation of lung cancer invading the chest wall of the apex

	Cases	30-day mortality		Hospital mortality
		Hospital	After discharge	
15. Operation of lung cancer invading the chest wall of the apex	610	0	0	2 (0.3)

(), Mortality %

Includes tumors invading the anterior apical chest wall and posterior apical chest wall (superior sulcus tumor, so called Pancoast type)

**Table 33:** Diagnostic procedures

	Cases	30-day mortality		Hospital mortality
		Hospital	After discharge	
Mediastinoscopic biopsy	188	3 (1.6)	0	5 (2.7)
Lung biopsy for diffuse parenchymal lung disease	627	3 (0.5)	0	6 (1.0)
Biopsy for lymph node, tumor and pleura	2859	31 (1.1)	24 (0.8)	43 (1.5)
Others	1492	49 (3.3)	10 (0.7)	110 (7.4)

(), Mortality %

### (C) Esophageal surgery

In 2018, the data collection method for esophageal surgery had been modified from self-reports using questionnaire sheets following each institution belonging to the Japanese Association for Thoracic Surgery to an automatic package downloaded from the NCD in Japan. Consequently, the registry excluded data for non-surgical cases with esophageal diseases. Furthermore, data regarding the histological classification of malignant tumors, multiple primary cancers, and mortality rates for cases with combined resection of other organs could not be registered because they were not included in the NCD. Instead, detailed data regarding postoperative surgical and non-surgical complications were collected from the NCD.

**Fig. 3:**
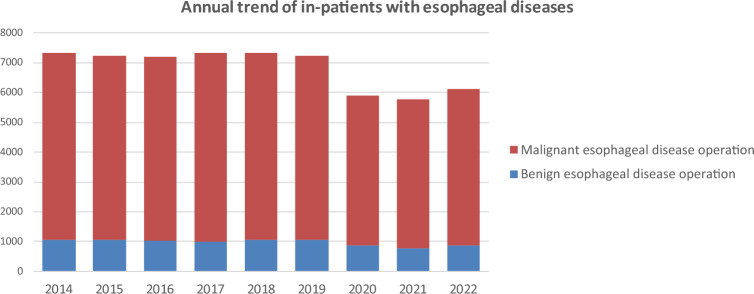
Annual trend of in-patients with esophageal diseases

Throughout 2022, 6132 patients underwent surgery for esophageal diseases (887 and 5245 for benign and malignant esophageal diseases, respectively) from institutions across Japan. Compared to 2019, there was a total decrease of 1103 cases (15.2%) observed. These significant declines which were largely influenced by the COVID-19 pandemic that began in 2020, with factors such as surgical restrictions, reduced medical visits, and postponed screenings being considered as contributing factors (Fig. 3). However, the number of esophageal surgeries in 2022 increased by 377 compared to 2021. As the issues related to COVID-19 are being resolved, a gradual recovery in the number of surgeries is expected in the future.

Concerning benign esophageal diseases (Table 34), thoracoscopic and/or laparoscopic surgeries were performed in 96.5% (83/86), 84.6% (391/462), 95.7% (45/47), and 29.2% (50/171) of patients with esophagitis (including esophageal ulcer), hiatal hernia, benign tumors, and achalasia, respectively. The decrease in the proportion of thoracoscopic and/or laparoscopic surgeries for achalasia is likely due to the gradual adoption of peroral endoscopic myotomy (POEM) in Japan. Conversely, 100% (85/85) of patients with spontaneous rupture of the esophagus underwent open surgery. Hospital mortality rates within 30 postoperative days were 0.6% (3/462), 3.5% (3/85) for hiatal hernia and spontaneous rupture of the esophagus, respectively.

**Table 34:** Benign esophageal diseases

	Operation ( +)				T/L*3			
	Cases	Hospital mortality			Cases	Hospital mortality		
		~ 30 days	31–90 days	Total (including after 91 days mortality)		~ 30 days	31–90 days	Total (including after 91 days mortality)
1.Achalasia	171	0	0	0	50	0	0	0
2.Benign tumor	47	0	0	0	45	0	0	0
3.Diverticulum	34	0	0	0	7	0	0	0
4.Hiatal hernia	462	3 (0.6)	1 (0.2)	4 (0.9)	391	0	0	0
5.Spontaneous rupture of the esophagus	85	3 (3.5)	4 (4.7)	7 (8.2)	0	0	0	0
6.Esophago-tracheal fistula	2	0	0	0	0	0	0	0
7.Esophagitis, esophageal ulcer	86	0	0	0	83	0	0	0
Total	887	6 (0.7)	5 (0.6)	11 (1.2)	576	0	0	0

(), Mortality %

*T/L* thoracoscopic and / or laparoscopic

The most common tumor location for malignant esophageal diseases was the thoracic esophagus (Table 35). Among the cases with esophageal malignancies, esophagectomy for superficial and advanced cancers was performed in 2007 (38.3%) and 3238 (61.7%), respectively. Hospital mortality rates within 30 days after esophagectomy were 0.4% and 1.0% for patients with superficial and advanced cancer, respectively.

**Table 35:** Malignant esophageal disease

		Operation( +)			Thoracoscopic and/or laparscopic procedure				
	Cases	Hospital mortality			Cases	Conversion to thoracotomy	Hospital mortality		
		~ 30 days	31–90 days	Total (including after 91days mortality)			~ 30days	31–90 days	Total (including after 91days mortality)
Location									
(1) Cervical esophagus	125	1 (0.8)	4 (3.2)	5 (4.0)	71	1 (1.4)	0	1 (1.4)	1 (1.4)
(2) Thoracic esophagus	4439	34 (0.8)	17 (0.4)	51 (1.1)	4157	22 (0.5)	31 (0.7)	15 (0.4)	46 (1.1)
(3) Abdominal esophagus	432	3 (0.7)	1 (0.2)	4 (0.9)	372	2 (0.5)	2 (0.5)	1 (0.3)	3 (0.8)
Total	4996	38 (0.8)	22 (0.4)	60 (1.2)	4600	25 (0.5)	33 (0.7)	17 (0.4)	50 (1.1)
Tumor depth									
(A) Superficial cancer(T1)									
(1) Transhiatal esophagectomy	7	0	0	0	0	0	0	0	0
(2) Mediastinoscopic esophagectomy and reconstruction	85	2 (2.4)	0	2 (2.4)	85	0	2 (2.4)	0	2 (2.4)
(3) Transthoracic (rt.) esophagectomy and reconstruction	1219	4 (0.3)	3 (0.2)	7 (0.6)	1139	5 (0.4)	4 (0.4)	3 (0.3)	7 (0.6)
(4) Transthoracic (lt.) esophagectomy and reconstruction	12	0	0	0	7	0	0	0	0
(5) Cervical esophageal resection and reconstruction	14	0	0	0	0	0	0	0	0
(6) Robot-assisted esophagectomy and reconstruction	525	2 (0.4)	2 (0.4)	4 (0.8)	524	1 (0.2)	2 (0.4)	2 (0.4)	4 (0.8)
(7) Others	17	0	0	0	0	0	0	0	0
(8) Esophagectomy without reconstruction	128	1 (0.8)	0	1 (0.8)	53	0	0	0	0
Subtotal	2007	9 (0.4)	5 (0.2)	14 (0.7)	1808	6 (0.3)	8 (0.4)	5 (0.3)	13 (0.7)
(B) Advanced cancer (T2–T4)									
(1) Transhiatal esophagectomy	13	1 (7.7)	0	1 (7.7)	0	0	0	0	0
(2) Mediastinoscopic esophagectomy and reconstruction	142	2 (1.4)	1 (0.7)	3 (2.1)	142	0	2 (1.4)	1 (0.7)	3 (2.1)
(3) Transthoracic (rt.) esophagectomy and reconstruction	2039	21 (1.0)	13 (0.6)	34 (1.7)	1852	18 (1.0)	18 (1.0)	9 (0.5)	27 (1.5)
(4) Transthoracic (lt.) esophagectomy and reconstruction	43	1 (2.3)	0	1 (2.3)	17	0	0	0	0
(5) Cervical esophageal resection and reconstruction	41	0	1 (2.4)	1 (2.4)	0	0	0	0	0
(6) Robot-assisted esophagectomy and reconstruction	825	5 (0.6)	2 (0.2)	7 (0.8)	825	1 (0.1)	5 (0.6)	2 (0.2)	7 (0.8)
(7) Others	14	0	0	0	0	0	0	0	0
(8) Esophagectomy without reconstruction	121	2 (1.7)	4 (3.3)	6 (5.0)	100	26 (26.0)	0	0	0
Subtotal	3238	32 (1.0)	21 (0.6)	53 (1.6)	2936	45 (1.5)	25 (0.9)	12 (0.4)	37 (1.3)
Total	5245	41 (0.8)	26 (0.5)	67 (1.3)	4744	51 (1.1)	33 (0.7)	17 (0.4)	50 (1.1)

	Cases	Overall morbidity	Morbidity ≥ CD III	Surgical complications						
				Surgical site infection			Anastomotic leakage	Recurrent nerve palsy		Wound dehiscence
				Superficial incision	Deep incision	Organ space				
Location										
(1) Cervical esophagus	125	77 (61.6)	35 (28.0)	11 (8.8)	9 (7.2)	15 (12.0)	17 (13.6)	16 (12.8)		3 (2.4)
(2) Thoracic esophagus	4439	2443 (55.0)	966 (21.8)	283 (6.4)	121 (2.7)	346 (7.8)	526 (11.8)	620 (14.0)		32 (0.7)
(3) Abdominal esophagus	432	207 (47.9)	75 (17.4)	19 (4.4)	9 (2.1)	30 (6.9)	44 (10.2)	43 (10.0)		4 (0.9)
Total	4996	2727 (54.6)	1076 (21.5)	313 (6.3)	139 (2.8)	391 (7.8)	587 (11.7)	679 (13.6)		39 (0.8)
Tumor depth										
(A) Superficial cancer(T1)										
(1) Transhiatal esophagectomy	7	6 (85.7)	3 (42.9)	1 (14.3)	0	1 (14.3)	1 (14.3)	2 (28.6)	0	
(2) Mediastinoscopic esophagectomy and reconstruction	85	52 (61.2)	15 (17.6)	6 (7.1)	5 (5.9)	7 (8.2)	14 (16.5)	23 (27.1)	1 (1.2)	
(3) Transthoracic (rt.) esophagectomy and reconstruction	1219	635 (52.1)	227 (18.6)	71 (5.8)	31 (2.5)	88 (7.2)	146 (12.0)	141 (11.6)	6 (0.5)	
(4) Transthoracic (lt.) esophagectomy and reconstruction	12	3 (25.0)	1 (8.3)	0	0	1 (8.3)	0	1 (8.3)	0	
(5) Cervical esophageal resection and reconstruction	14	9 (64.3)	6 (42.9)	1 (7.1)	2 (14.3)	3 (21.4)	2 (14.3)	2 (14.3)	0	
(6) Robot-assisted esophagectomy and reconstruction	525	289 (55.0)	130 (24.8)	35 (6.7)	11 (2.1)	47 (9.0)	73 (13.9)	68 (13.0)	3 (0.6)	
(7) Others	17	6 (35.3)	0	0	0	0	1 (5.9)	0	0	
(8) Esophagectomy without reconstruction	128									
Subtotal	2007	1000 (49.8)	382 (19.0)	114 (5.7)	49 (2.4)	147 (7.3)	237 (11.8)	237 (11.8)	10 (0.5)	
(B) Advanced cancer (T2–T4)										
(1) Transhiatal esophagectomy	13	8 (61.5)	6 (46.2)	2 (15.4)	1 (7.7)	2 (15.4)	4 (30.8)	1 (7.7)	1 (7.7)	
(2) Mediastinoscopic esophagectomy and reconstruction	142	87 (61.3)	40 (28.2)	14 (9.9)	8 (5.6)	10 (7.0)	20 (14.1)	35 (24.6)	0	
(3) Transthoracic (rt.) esophagectomy and reconstruction	2039	1109 (54.4)	430 (21.1)	112 (5.5)	48 (2.4)	160 (7.8)	216 (10.6)	262 (12.8)	21 (1.0)	
(4) Transthoracic (lt.) esophagectomy and reconstruction	43	20 (46.5)	9 (20.9)	3 (7.0)	1 (2.3)	4 (9.3)	6 (14.0)	2 (4.7)	0	
(5) Cervical esophageal resection and reconstruction	41	25 (61.0)	10 (24.4)	2 (4.9)	1 (2.4)	1 (2.4)	3 (7.3)	4 (9.8)	0	
(6) Robot-assisted esophagectomy and reconstruction	825	468 (56.7)	196 (23.8)	66 (8.0)	29 (3.5)	63 (7.6)	97 (11.8)	138 (16.7)	7 (0.8)	
(7) Others	14	10 (71.4)	3 (21.4)	0	2 (14.3)	4 (28.6)	4 (28.6)	0	0	
(8) Esophagectomy without reconstruction	121									
Subtotal	3238	1727 (53.3)	694 (21.4)	199 (6.1)	90 (2.8)	244 (7.5)	350 (10.8)	442 (13.7)	29 (0.9)	
Total	5245	2727 (52.0)	1076 (20.5)	313 (6.0)	139 (2.7)	391 (7.5)	587 (11.2)	679 (12.9)	39 (0.7)	

	Cases	Nonsurgical complications										Readmission within 30d	Reoperation within 30d
		Pneumonia	Unplanned intubation	Prolonged ventilation > 48 h		Pulmonary embolism	Atelectasis	Renal failure	CNS events	Cardiac events	Septic shock		
Location													
(1) Cervical esophagus	125	20 (16.0)	5 (4.0)	15 (12.0)		0	5 (4.0)	0	1 (0.8)	0	1 (0.8)	2 (1.6)	22 (17.6)
(2) Thoracic esophagus	4439	740 (16.7)	175 (3.9)	149 (3.4)		47 (1.1)	216 (4.9)	18 (0.4)	18 (0.4)	15 (0.3)	31 (0.7)	113 (2.5)	260 (5.9)
(3) Abdominal esophagus	432	59 (13.7)	15 (3.5)	13 (3.0)		3 (0.7)	22 (5.1)	2 (0.5)	0	0	3 (0.7)	9 (2.1)	23 (5.3)
Total	4996	819 (16.4)	195 (3.9)	177 (3.5)		50 (1.0)	243 (4.9)	20 (0.4)	19 (0.4)	15 (0.3)	35 (0.7)	124 (2.5)	305 (6.1)
Tumor depth													
(A) Superficial cancer (T1)													
(1) Transhiatal esophagectomy	7	1 (14.3)	1 (14.3)	1 (14.3)		0	0	0	0	0	1 (14.3)	0	2 (28.6)
(2) Mediastinoscopic esophagectomy and reconstruction	85	12 (14.1)	4 (4.7)	4 (4.7)		0	2 (2.4)	0	0	0 (0.0)	0	1 (1.2)	2 (2.4)
(3) Transthoracic (rt.) esophagectomy and reconstruction	1219	171 (14.0)	42 (3.4)	31 (2.5)		13 (1.1)	57 (4.7)	1 (0.1)	5 (0.4)	5 (0.4)	6 (0.5)	29 (2.4)	69 (5.7)
(4) Transthoracic (lt.) esophagectomy and reconstruction	12	0	0	0		0	0	4 (33.3)	0	0	0	0	1 (8.3)
(5) Cervical esophageal resection and reconstruction	14	1 (7.1)	2 (14.3)	2 (14.3)		0	1 (7.1)	0	1 (7.1)	0	0	0	6 (42.9)
(6) Robot-assisted esophagectomy and reconstruction	525	93 (17.7)	19 (3.6)	14 (2.7)		10 (1.9)	27 (5.1)	0	3 (0.6)	2 (0.4)	1 (0.2)	20 (3.8)	28 (5.3)
(7) Others	17	0	0	0		0	0	0	0	0	0	1 (5.9)	0
(8) Esophagectomy without reconstruction	128												0
Subtotal	2007	278 (13.9)	68 (3.4)	52 (2.6)		23 (1.1)	87 (4.3)	5 (0.2)	9 (0.4)	7 (0.3)	8 (0.4)	51 (2.5)	108 (5.4)
(B) Advanced cancer (T2–T4)													
(1) Transhiatal esophagectomy	13	1 (7.7)	2 (15.4)	0		0	0	0	0	0	1 (7.7)	0	2 (15.4)
(2) Mediastinoscopic esophagectomy and reconstruction	142	27 (19.0)	10 (7.0)	9 (6.3)		0	4 (2.8)	0	1 (0.7)	1 (0.7)	5 (3.5)	4 (2.8)	15 (10.6)
(3) Transthoracic (rt.) esophagectomy and reconstruction	2039	358 (17.6)	87 (4.3)	88 (4.3)		20 (1.0)	113 (5.5)	8 (0.4)	4 (0.2)	6 (0.3)	15 (0.7)	46 (2.3)	116 (5.7)
(4) Transthoracic (lt.) esophagectomy and reconstruction	43	7 (16.3)	3 (7.0)	2 (4.7)		1 (2.3)	3 (7.0)	0	0	0	0	1 (2.3)	3 (7.0)
(5) Cervical esophageal resection and reconstruction	41	4 (9.8)	1 (2.4)	3	(7.3)	0	1 (2.4)	0	1 (2.4)	0	0	0	5 (12.2)
(6) Robot-assisted esophagectomy and reconstruction	825	141 (17.1)	24 (2.9)	23 (2.8)		6 (0.7)	35 (4.2)	7 (0.8)	4 (0.5)	1 (0.1)	6 (0.7)	20 (2.4)	56 (6.8)
(7) Others	14	3 (21.4)	0	0		0	0	0	0	0	0	2 (14.3)	0
(8) Esophagectomy without reconstruction	121												
Subtotal	3238	541 (16.7)	127 (3.9)	125 (3.9)		27 (0.8)	156 (4.8)	15 (0.5)	10 (0.3)	8 (0.2)	27 (0.8)	73 (2.3)	197 (6.1)
Total	5245	819 (15.6)	195 (3.7)	177 (3.4)		50 (1.0)	243 (4.6)	20 (0.4)	19 (0.4)	15 (0.3)	35 (0.7)	124 (2.4)	305 (5.8)

**Fig. 4:**
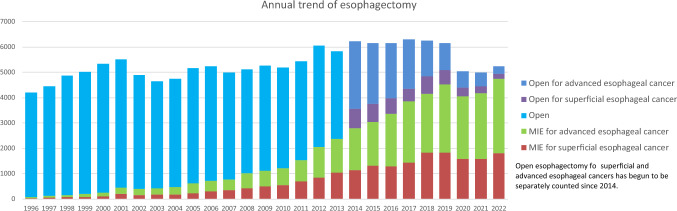
Annual trend of esophagectomy

Among esophagectomy procedures, transthoracic esophagectomy via right thoracotomy or right thoracoscopy was most commonly adopted for patients with superficial (1219/2007, 60.7%) and advanced cancer (2039/3238, 63.0%) (Table 35). Transhiatal esophagectomy, which is commonly performed in Western countries, was adopted in only 7 (0.3%) and 13 (0.4%) patients with superficial and advanced cancer who underwent esophagectomy in Japan, respectively. Minimally invasive esophagectomy (MIE) including thoracoscopic and/or laparoscopic esophagectomy, robot-assisted esophagectomy and mediastinoscopic esophagectomy was utilized in 1808 (90.1%) and 2936 (90.7%) patients with superficial and advanced cancer, respectively. Incidence of MIE for superficial or advanced cancer have been increasing, whereas that of open surgery, especially for advanced cancer, has been decreasing annually (Fig. 4). Although mediastinoscopic esophagectomy was performed only for 85 (4.2%) and 142 (4.4%) patients with superficial and advanced esophageal cancer, respectively. Robot-assisted esophagectomy has been remarkably increased since 2018 when the insurance approval was obtained in Japan, and performed for 525 (26.2%) and 825 (25.5%) patients with superficial and advanced esophageal cancer, respectively in 2021 [4]. Patients who underwent robot-assisted surgery are increasing for both superficial and advancer esophageal cancers (23.8% and 28.1% increases compared to that in 2021, respectively). Hospital mortality rates within 30 days after MIE were 0.4% and 0.9% for patients with superficial and advanced cancer, respectively (Table 35).

Detailed data collection regarding postoperative surgical and non-surgical complications was initiated in 2018. Overall, 1076 (20.5%) of 5245 patients developed grade III or higher complications based on the Clavien–Dindo classification in 2022 (Table 35). The incidence of grade III or higher complications was relatively higher in cervical esophageal cancer compared to thoracic or abdominal esophageal cancer. Among surgical complications in patients with advanced esophageal cancer, anastomotic leakage and recurrent nerve palsy occurred in 10.6% and 12.8% of the patients who underwent right transthoracic esophagectomy, in 11.8% and 16.7% of those who underwent robot-assisted esophagectomy, and in 14.1% and 24.6% of those who underwent mediastinoscopic esophagectomy, respectively. Among non-surgical postoperative complications, pneumonia occurred in 16.4% of the patients, 3.9% of whom underwent unplanned intubation. Postoperative pulmonary embolism occurred in 1.0% of the patients. These complication rates, including the others, were similar to those in 2021.

We aim to continue our efforts in collecting comprehensive survey data through more active collaboration with the Japan Esophageal Society and other related institutions, with caution due to the impact of COVID-19 pandemic.

## Data Availability

Based on the data use policy of JATS, data access is approved through assessment by the JATS: Committee for Scientific Affairs. Those interested in using the data should contact the JATS: Committee for Scientific Affairs(survey@jpats.org) to submit a proposal. The use of the data is granted for the approved study proposals.
